# Urea Derivative Catalyzed Enantioselective Hydroxyalkylation of Hydroxyindoles with Isatins

**DOI:** 10.3390/molecules24213944

**Published:** 2019-10-31

**Authors:** Hao Wu, Liming Wang, Junwei Zhang, Ying Jin

**Affiliations:** Department of Pharmacy, Jilin Medical University, Jilin 132013, China; wuhao931230@163.com (H.W.); 13630635312@163.com (L.W.); 15144215202@163.com (J.Z.)

**Keywords:** urea derivatives, enantioselective, hydroxyalkylation, hydroxyindoles, isatins

## Abstract

The enantioselective transformations of indoles preferentially take place in the more-reactive azole ring. However, the methods for the enantioselective functionalization of the indole benzene ring are scarce. In this paper, a series of bifunctional (thio)urea derivatives were used to organocatalyze the enantioselective Friedel-Crafts hydroxyalkylation of indoles with isatins. The resulting products were obtained in good yields (65–90%) with up to 94% enantiomer excess (ee). The catalyst type and the substrate scope were broadened in this methodology.

## 1. Introduction

The indole scaffolds are privileged skeletons, as they have been widely found in many bioactive natural products, pharmaceuticals, and material molecules [1,2,3,4,5,6]. The synthesis and modification of indoles have attracted intensive interest for a long time. Accordingly, the enantioselective functionalization of indoles has been one of the most studied reactions in asymmetric catalysis [7,8,9,10,11,12,13,14]. Indoles show a high nucleophilic reactivity in the azole ring, which preferentially reacts with electrophilic aromatic substitution at the C-3 position [15,16,17,18,19,20,21,22]. Additionally, these Friedel-Crafts (F-C) reactions also selectively take place at the positions C-2 [23,24,25,26,27,28,29,30] and N-1 [31,32,33,34,35,36,37] by using different strategies. However, the functionalization in the benzene ring of indole is still difficult, which generally requires the presence of directing or blocking group in the azole ring [38,39,40,41,42,43,44,45,46] and employs transition metal catalysts [47,48,49].

In 2015, Jørgensen reported the first example of catalytic asymmetric F-C alkylation of 4-hydroxyindole occurring at the C-5 position [50]. Subsequently, Pedro and coworkers developed the first asymmetric F-C reaction of hydroxyindoles with isatin-derived ketimines [51] or isatins [52] organocatalyzed by *cinchona*-derived squaramide. These methodologies allow the functionalization of indoles in every position of the benzene ring in a regioselective and enantioselective fashion, by utilizing the activating/directing ability of hydroxyl group. Moreover, the OH group was removed smoothly upon hydrogenolysis of the corresponding triflate. Very recently, Zhao [53] and Deng [54], respectively, reported the *cinchona*-derived squaramide organocatalyzed the enantioselective F-C transformation in the benzene ring of indoles, employing other electrophiles with hydroxyindoles. In particular, the resulting hydroxyindole moiety is of high importance in medicinal chemistry and natural products [51,55,56,57], showing great potential in diversity-oriented synthesis and drug discovery.

**Figure 1:**
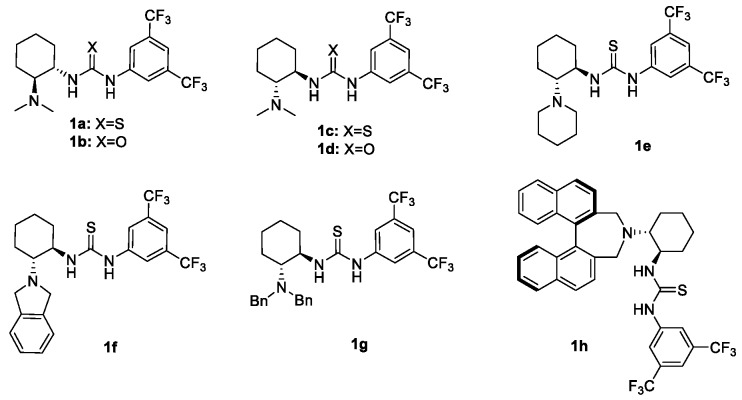
(Thio)urea derivatives screened as organocatalysts (1a–1h).

In spite of the significant developments, there is a high demand for the enantioselective functionalization in the benzene ring of indole by using new types of catalysts. Takemoto’s catalyst is the commercially available chiral organocatalyst, which was first synthesized by Takemoto in 2003 [58]. Then, they were used efficiently for a wide range of diastereoselective and enantioselective reactions [59,60,61,62,63,64,65]. We herein first reported the enantioselective hydroxyalkylation of hydroxyindoles with isatins by employing Takemoto-type catalysts 1a–1h bearing (thio)urea-tertiary amine moiety (Figure 1).

## 2. Results and Discussion

According to the optimized conditions reported by Pedro [52], we first screened the bifunctional catalysts in the reaction of 4-hydroxyindole and isatin (2a) with Et_2_O as a solvent in the presence of 10 mol% of (thio)urea catalysts 1a–1h (Table 1). Initially, widely used thiourea 1a was examined at room temperature. However, the desired 5-alkylated indole 3a was afforded in 70% yield with 68%ee (entry 1). Gratifyingly, when (*S*, *S*)-urea catalyst 1b was used to induce the reaction, the obviously improved yield and ee value were observed (entry 2). Based on a comparison of the optical rotation of the product with a value from the literature [52], the absolute configuration of the major product was determined to be *R*. The enantiomeric (*R*, *R*)-urea catalyst 1d gave the *S* major isomer with slightly decreased ee of 76% (entry 4). Surprisingly, piperidine-based thiourea 1e could not catalyze the reaction. Moreover, the further endeavor to improve ee by increasing the steric bulk of the basic moiety of bifunctional catalysts 1f–1h was not successful (entries 6–8). Therefore, the catalyst 1b, containing *N*, *N*-dimethyl tertiary amine and urea moiety, showed the best yield and enantioselectivity.

To optimize the enantioselectivity of the transformation, we investigated a variety of different reaction conditions (Table 2). The survey of solvents showed that Et_2_O was the optimal solvent in terms of the yield and enantioselectivity (entries 1–4). The screening of catalyst loading exhibited that 10 mol% equivalent of 1b was optimal 5 mol% loading of catalyst led to reduction both in the yield and ee (entry 6), and 20 mol% loading offered no improvement in the asymmetric induction, albeit with a slightly improved yield (entry 7 vs. entry 1). When the reaction temperature was lowered from rt to 0 °C, an improved ee of 83% was afforded (entry 8 vs. entry 1). The further temperature decreases to −20 °C and −40 °C were detrimental for both yield and enantiocontrol (entries 10, 11 vs. entry 8). Furthermore, diluting the reaction concentration by half produced a slightly lower ee value and decreased yield (entry 5). Based on these experiments, the optimized conditions were determined to be Et_2_O as the solvent with a 10 mol% loading of catalyst 1b at 0 °C.

With the optimized conditions in hand, the substrate scope of this protocol was investigated. As shown in Table 3, the corresponding 5-alkylated products were obtained in good yield (65–90%) with >20:1 regioselectivities determined by ^1^H NMR. A wide range of isatins bearing various substituents on the phenyl ring such as halogens, methyl and methoxyl were tolerated in good to excellent yields with 71–94%ee except 4-bromo substituted isatin which produced 3b with only 50%ee in low yield (entry 2). It might be caused by the adjacent substituent to the carbonyl of isatin. Therefore, the enantioselectivities were obviously affected by the substituted position on the phenyl ring of isatins. The 5- and 7-substituted isatins appeared to favor higher enantioselectivities (entries 3–6 and entries 8–9). In the case of *N*-methylisatin and *N*-benzylisatin, moderate ee value was obtained (entry 10–11). Of all the different substrates listed in Table 3, the reaction of 5-Cl-substituted isatin afforded the optimal enantiomeric excess (94%ee, entry 5). Compared with the results of the reported reference [52], the similar enantioselectivities were achieved with *N*-unsubstituted isatins and *N*-Methylisatin as the reactants (entries 1, 6, and 10), whereas the reaction of *N*-benzylisatin with 4-Hydroxyindole got the significantly decreased ee value (entry 11). However, the obviously high yields were observed with *N*-unsubstituted isatins as the substrate (entries 1, 6). The reported literature [52] mainly focused on the reaction of *N*-benzylisatins and only tried three isatins containing the free NH group. We broadened the substrate scope by investigating nine *N*-unsubstituted isatins.

**Figure 2:**
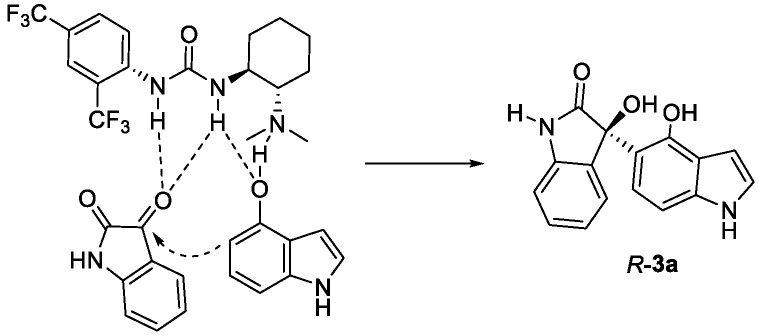
Proposed stereochemical model.

Based on the obtained absolute configuration described above and previously reported enantioselective organocatalytic reactions of isatin-derived ketimines [51], a proposed transition-state model is depicted in Figure 2. Both hydroxyindole and isatin are activated through hydrogen bonding with bifunctional urea catalyst 1b. Then, the reaction proceeds with a *Re*-face addition of hydroxyindoles to isatin 2a, affording the desired product *R*-3a.

**Scheme 1:**
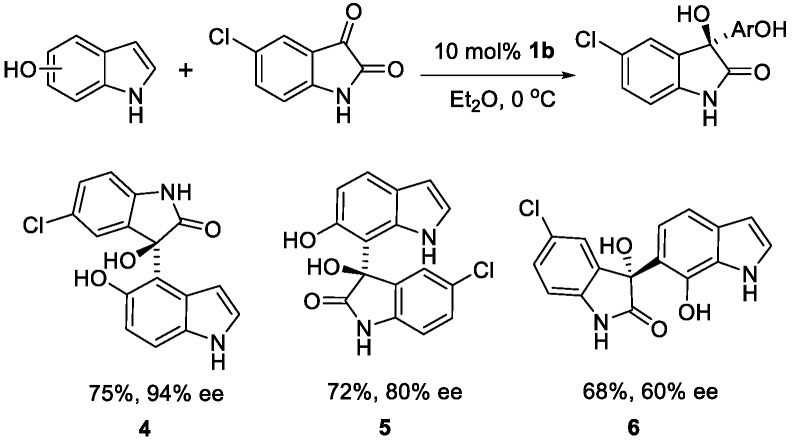
Scope for the enantioselective hydroxyalkylation between different hydroxyindoles and isatin. Reaction conditions: hydroxyindole (0.1 mmol), isatin (0.1 mmol), 1b (10 mol %) in Et_2_O (1.5 mL) at 0 °C. Isolated yields after column chromatography. >20:1 regioselectivity determined by ^1^H NMR.

In order to achieve the functionalization of every position in the carbocyclic ring, we respectively examined the reaction of 5-, 6-, and 7-hydroxyindole and 5-Cl substituted isatin (Scheme 1). As we expected, the corresponding alkylated indoles were isolated with excellent regioselectivity in all cases. The 5-hydroxyindole showed optimal reactivity (75% yield) and enantioselectivity (94%ee) under the optimized reaction conditions to give a 4-alkylated product. In a similar manner, 6-hydroxyindole was functionalized selectively in the C-7 position with good enantioselectivity (80%ee). When we tried the reaction of 7-hydroxyindole, the 6-alkylated product was obtained with low enantioselectivity (60%ee).

## 3. Conclusion

In summary, we described how the first urea derivative catalyst promoted the enantioselective hydroxyalkylation of hydroxyindoles with isatins. The enantioselective modification happened in the benzene ring rather than in the azole ring to give the desired hydroxyalkylated indoles with high enantioselectivity (up to 94%ee). Furthermore, we used our optimized conditions to expand upon the substrate scope of this transformation.

## 4. Experimental

### 4.1. Chemistry

The ^1^H NMR spectra were recorded on a 500 MHz spectrometer, using CD_3_OD–*d*_4_ as a solvent. The chemical shifts were reported in ppm, and the residual CD_3_OD signal was used as a reference (3.31 and 4.87 ppm). The splitting patterns of the signals were reported as s, singlet; d, doublet; t, triplet; q, quartet; dd, doublet of doublets; and m, multiplet. The ^13^C NMR spectra were recorded on a 125 MHz instrument using CD_3_OD–*d*_4_ as a solvent. The chemical shifts were reported in ppm, and the residual CD_3_OD signal was used as a reference (49.0 ppm). High-resolution mass spectra (HRMS) were measured on a triple TOF 5600+ mass spectrometer equipped with an electrospray ionization (ESI) source in the negative-ion mode. The enantiomeric excess (ee) values of the products were determined by chiral HPLC, using Daicel Chiralpak AD-H columns (4.6 mm*250 mm). The reactions were monitored by thin layer chromatography (TLC). Purifications by column chromatography were conducted over silica gel (200–300 mesh). The organocatalysts 1a–h were purchased from Daicel chiral technologies (China) company.

### 4.2. General Procedure for the Enantioselective Friedel-Crafts Reaction of Hydroxyindole and Isatins

To a tube containing hydroxyindole (13.3 mg, 0.1 mmol) and isatin (2, 23.7, 0.1 mmol) and catalyst 1b (4.0 mg, 0.01 mmol), Et_2_O (1.5 mL) was added. The resulting mixture was stirred at room temperature for 7 h (TLC). After the reaction was finished, the reaction directly poured into a column chromatography on silica gel with hexane/EtOAc (3:1) as eluent to afford the products 3a–k. Experimental data can be found in Supplementary Materials.

(+)-(*R*)-3-Hydroxy-3-(4-hydroxy-1*H*-indol-5-yl)indolin-2-one (**3a**): brown oil (85% yield); ^1^H NMR (500 MHz, CD_3_OD) *δ* 7.28–7.19 (m, 2H), 7.10–7.05 (m, 1H), 7.01 (td, *J* = 7.5, 1.0 Hz, 1H), 6.93 (d, *J* = 8.0 Hz, 1H), 6.82 (d, *J* = 1.0 Hz, 2H), 6.53 (d, *J* = 3.0 Hz, 1H) ppm; HRMS (ESI) *m*/*z*: HRMS (ESI): calcd for C_16_H_11_N_2_O_3_^−^ [M-1]^−^: 279.0775, found: 279.0770; [α]_D_^25^ = +46.85 (c 0.5, MeOH). Enantiomeric excess (83%) was determined by chiral HPLC (ChiralpakAD-H), hexane:^i^PrOH = 80:20, 1.5 mL/min, major enantiomer t_R_ = 14.1 min, minor enantiomer t_R_ = 17.3 min.

(+)-(*S*)-4-bromo-3-hydroxy-3-(4-hydroxy-1*H*-indol-5-yl)indolin-2-one (**3b**): brown oil (72% yield); ^1^H NMR (500 MHz, CD_3_OD) *δ* 7.16 (t, *J* = 8.0 Hz, 1H), 7.11 (d, *J* = 8.5 Hz, 1H), 7.07 (d, *J* = 3.5 Hz, 1H), 6.91 (dd, *J* = 7.5, 1.0 Hz, 1H), 6.82 (d, *J* = 8.0 Hz, 1H), 6.50 (d, *J* = 3.0 Hz, 1H); ^13^C NMR (125 MHz, CD_3_OD) *δ* 181.8, 150.2, 145.6, 139.3, 132.9, 131.9, 127.8, 124.1, 122.3, 121.3, 120.1, 110.1, 103.8, 99.7, 99.6, 91.5; HRMS (ESI): calcd for C_16_H_10_BrN_2_O_3_^−^ [M-1]^−^: 356.9880, found: 356.9874; [α]_D_^25^ = +5.10 (c 0.3, MeOH). Enantiomeric excess (50%) was determined by chiral HPLC (Chiralpak AD-H), hexane:^i^PrOH = 80:20, 1.5 mL/min, major enantiomer t_R_ = 13.2 min, minor enantiomer t_R_ = 17.3 min.

(+)-(*R*)-5-methyl-3-hydroxy-3-(4-hydroxy-1*H*-indol-5-yl)indolin-2-one (**3c**): brown oil (85% yield); ^1^H NMR (500 MHz, CD_3_OD) *δ* 7.08–7.05 (m, 3H), 6.83–6.80 (m, 2H), 6.78 (d, *J* = 8.5 Hz, 1H), 6.53 (d, *J* = 3.0 Hz, 1H), 2.26 (s, 3H); ^13^C NMR (125 MHz, CD_3_OD) *δ* 182.7, 150.5, 140.6, 139.4, 134.7, 133.4, 130.7, 126.9, 124.4, 121.7, 120.4, 114.1, 110.9, 103.9, 99.7, 80.7, 21.1; HRMS (ESI): calcd for C_17_H_13_N_2_O_3_^−^ [M-1]^−^: 293.0932, found: 293.0927; [α]_D_^25^ = +64.42 (c 0.60, MeOH). Enantiomeric excess (85%) was determined by chiral HPLC (Chiralpak AD-H), hexane:^i^PrOH = 80:20, 1.5 mL/min, major enantiomer t_R_ = 12.8 min, minor enantiomer t_R_ = 14.3 min.

(+)-(*R*)-5-methoxyl-3-hydroxy-3-(4-hydroxy-1*H*-indol-5-yl)indolin-2-one (**3d**): brown oil (78% yield); ^1^H NMR (500 MHz, CD_3_OD) *δ* 7.09 (d, *J* = 3.5 Hz,1H), 6.87–6.78 (m, 5H), 6.53 (d, *J* = 3.0 Hz, 1H), 3.71 (s, 3H). ^13^C NMR (125 MHz, CD_3_OD) *δ* 182.6, 157.6, 150.5, 139.5, 136.3, 135.9, 124.4, 121.7, 120.4, 115.4, 114.0, 112.8, 111.7, 104.0, 99.8, 81.0, 56.2; HRMS (ESI): calcd for C_17_H_13_N_2_O_4_^−^[M-1]^−^: 309.0881, found: 309.0870; [α]_D_^25^ = +38.52 (c 0.65, MeOH). Enantiomeric excess (82%) was determined by chiral HPLC (Chiralpak AD-H), hexane:^i^PrOH = 80:20, 1.5 mL/min, minor enantiomer t_R_ = 20.8 min, major enantiomer t_R_ = 22.6 min.

(+)-(*R*)-5-chloro-3-hydroxy-3-(4-hydroxy-1*H*-indol-5-yl)indolin-2-one (**3e**): brown oil (81% yield); ^1^H NMR (500 MHz, CD_3_OD) *δ* 7.22 (dd, *J* = 8.0, 2.0 Hz, 1H), 7.13 (d, *J* = 3.0 Hz, 1H), 7.09 (d, *J* = 3.5 Hz, 1H), 7.03 (d, *J* = 8.5 Hz, 1H), 6.90 (dd, *J* = 13.5, 8.5 Hz, 2H), 6.53 (d, *J* = 3.0 Hz, 1H); ^13^C NMR (125 MHz, CD_3_OD) *δ* 182.3, 149.6, 142.0, 139.5, 136.8, 130.1, 128.7, 126.1, 124.5, 121.3, 120.2, 114.3, 112.2, 104.1, 99.6, 79.7; HRMS (ESI): calcd for C_16_H_10_ClN_2_O_3_^−^ [M-1]^−^: 313.0385, found: 313.0380; [α]_D_^25^ = +90.51 (c 0.46, MeOH). Enantiomeric excess (94%) was determined by chiral HPLC (Chiralpak AD-H), hexane:^i^PrOH = 80:20, 1.5 mL/min, major enantiomer t_R_ = 13.4 min, minor enantiomer t_R_ = 14.8 min.

(+)-(*R*)-5-bromo-3-hydroxy-3-(4-hydroxy-1*H*-indol-5-yl)indolin-2-one (**3f**): brown oil (80% yield); ^1^H NMR (500 MHz, CD_3_OD) *δ* 7.37 (dd, *J* = 8.0, 3.0 Hz, 1H), 7.24 (d, *J* = 2.0 Hz, 1H), 7.09 (d, *J* = 3.0 Hz, 1H), 7.03 (d, *J* = 8.5 Hz, 1H), 6.91 (d, *J* = 8.5 Hz, 1H), 6.85 (d, *J* = 8.5 Hz, 1H), 6.51 (d, *J* = 2.5 Hz, 1H); HRMS (ESI): calcd for C1_6_H_10_BrN_2_O_3_^−^ [M-1]^−^: 356.9880, found: 356.9873; [α]_D_^25^ = +80.27 (c 0.67, MeOH). Enantiomeric excess (90%) was determined by chiral HPLC (Chiralpak AD-H), hexane:^i^PrOH = 80:20, 1.5 mL/min, major enantiomer t_R_ = 21.5 min, minor enantiomer t_R_ = 23.7 min.

(+)-(*R*)-6-bromo-3-hydroxy-3-(4-hydroxy-1*H*-indol-5-yl)indolin-2-one (**3g**): brown oil (83% yield); ^1^H NMR (500 MHz, CD_3_OD) *δ* 7.13 (dd, *J* = 8.0, 1.5 Hz, 1H), 7.09 (d, *J* = 1.5 Hz, 1H), 7.03 (d, *J* = 3.5 Hz, 2H), 6.88 (dd, *J* = 10.0, 7.5 Hz, 1H), 6.50 (d, *J* = 3.0 Hz, 1H). ^13^C NMR (125 MHz, CD_3_OD) *δ* 182.4, 149.6, 145.0, 139.6, 134.1, 127.4, 126.3, 124.4, 123.5, 121.3, 120.2, 114.4, 114.2, 104.0, 99.6, 79.3 ppm; HRMS (ESI): calcd for C_16_H_10_BrN_2_O_3_^−^ [M-1]^−^: 356.9880, found: 356.9873; [α]_D_^25^ = +8.73 (c 0.76, MeOH). Enantiomeric excess (71%) was determined by chiral HPLC (Chiralpak AD-H), hexane:^i^PrOH = 80:20, 1.5 mL/min, major enantiomer t_R_ = 13.0 min, minor enantiomer t_R_ = 17.2 min.

(+)-(*R*)-7-fluoro-3-hydroxy-3-(4-hydroxy-1*H*-indol-5-yl)indolin-2-one (**3h**): brown oil (88% yield); ^1^H NMR (500 MHz, CD_3_OD) *δ* 7.10–6.94 (m, 4H), 7.01 (d, *J* = 8.6 Hz, 1H), 6.95 (d, *J* = 8.0, 4.0 Hz, 1H), 6.89 (d, *J* = 8.5 Hz, 1H), 6.51 (d, *J* = 2.5 Hz, 1H); ^13^C NMR (125 MHz, CD_3_OD) δ 182.2, 149.6 (d, *J* = 59.5 Hz), 147.6, 139.5, 137.8 (d, *J* = 9.0 Hz), 130.4 (d, *J* = 49.5 Hz), 124.4, 124.2 (d, *J* = 22.0 Hz), 121.8 (d, *J* = 10.5 Hz), 121.4, 120.2, 117.0 (d, *J* = 69.5 Hz), 114.4, 104.0 (d, *J* = 25.5 Hz), 99.6 (d, *J* = 22.5 Hz), 79.8 (d, *J* = 7.5 Hz) ppm; HRMS (ESI): calcd for C_16_H_10_FN_2_O_3_^−^[M-1]^−^: 297.0681, found: 297.0677; [α]_D_^25^ = +43.06 (c 0.82, MeOH). Enantiomeric excess (92%) was determined by chiral HPLC (Chiralpak AD-H), hexane:^i^PrOH = 80:20, 1.5 mL/min, major enantiomer t_R_ = 10.5 min, minor enantiomer t_R_ = 12.7 min.

(+)-(*R*)-7-chloro-3-hydroxy-3-(4-hydroxy-1*H*-indol-5-yl)indolin-2-one (**3i**): brown oil (90% yield); ^1^H NMR (500 MHz, CD_3_OD) *δ* 7.22 (dd, *J* = 8.0, 1.0 Hz, 1H), 7.06 (dd, *J* = 7.5, 1.0 Hz, 2H), 7.02 (d, *J* = 8.5 Hz, 1H), 6.96–6.91 (m, 1H), 6.87 (d, *J* = 8.5 Hz, 1H), 6.48 (d, *J* = 2.5 Hz, 1H); ^13^C NMR (125 MHz, CD_3_OD) *δ* 182.2, 149.5, 141.2, 139.6, 136.7, 130.1, 124.5, 124.4, 124.3, 121.3, 120.1, 116.1, 114.5, 104.0, 99.6, 80.2; HRMS (ESI): calcd for C_16_H_10_ClN_2_O_3_^−^ [M-1]^−^: 313.0385, found: 313.0380; [α]_D_^25^ = +14.45 (c = 0.49, MeOH). Enantiomeric excess (83%) was determined by chiral HPLC (Chiralpak AD-H), hexane:^i^PrOH = 80:20, 1.5 mL/min, major enantiomer t_R_ = 11.2 min, minor enantiomer t_R_ = 14.7 min.

(+)-(*R*)-3-hydroxy-3-(4-hydroxy-1*H*-indol-5-yl)-1-methylindolin-2-one (**3j**): brown oil (82 % yield); ^1^H NMR (500 MHz, CD_3_OD) *δ* 7.31 (dt, *J* = 7.5, 1.0 Hz, 1H), 7.21 (d, *J* = 7.5 Hz, 1H), 7.07 (t, *J* = 4.0 Hz, 1H), 7.03 (dt, *J* = 7.5, 0.5 Hz, 1H), 6.97 (dd, *J* = 12.5, 8.0 Hz, 2H), 6.86 (dd, *J* = 8.5, 0.5 Hz, 1H), 6.50 (dd, *J* = 3.5, 1.0 Hz, 1H), 3.23 (s, 3H); HRMS (ESI): calcd for C_17_H_13_N_2_O_3_^−^ [M-1]^−^: 293.0932, found: 293.0922; [α]_D_^25^ = +52.23 (c = 0.90, MeOH). Enantiomeric excess (78%) was determined by chiral HPLC (Chiralpak AD-H), hexane:^i^PrOH = 80:20, 1.5 mL/min, major enantiomer t_R_ = 12.5 min, minor enantiomer t_R_ = 15.3 min.

(+)-(*R*)-1-benzyl-3-hydroxy-3-(4-hydroxy-1*H*-indol-5-yl)indolin-2-one (**3k**): colorless oil (87% yield); ^1^H NMR (500 MHz, CD_3_OD) *δ* 7.43 (d, *J* = 7.0 Hz, 2H), 7.34 (t, *J* = 7.5 Hz, 2H), 7.26 (t, *J* = 7.5 Hz, 1H), 7.19–7.15 (m, 2H), 7.09 (d, *J* = 3.0 Hz, 1H), 7.03–6.96 (m, 2H), 6.89 (dd, *J* = 8.5, 1.0 Hz, 1H), 6.80 (d, *J* = 8.0 Hz, 1H), 6.53 (dd, *J* = 3.5, 1.0 Hz, 1H), 5.02 (d, *J* = 16.0 Hz, 1H), 4.93 (d, *J* = 16.0 Hz, 1H); ^13^C NMR (125 MHz, CD_3_OD) *δ* 180.8, 149.6, 144.3, 139.6, 137.5, 134.3, 130.2, 129.8, 128.6, 128.4, 125.7, 124.4, 124.2, 121.5, 120.23, 114.7, 110.7, 104.0, 99.7, 79.5, 44.7 ppm; HRMS (ESI): calcd for C23H17N2O3^−^ [M-1]^−^: 369.1245, found: 369.1239; [α]_D_^25^ = +25.51 (c = 0.58, MeOH). Enantiomeric excess (74%) was determined by chiral HPLC (Chiralpak AD-H), hexane:^i^PrOH = 90:10, 1.5 mL/min, minor enantiomer t_R_ = 17.5 min, major enantiomer t_R_ = 19.8 min.

(-)-(*R*)-5-chloro-3-hydroxy-3-(5-hydroxy-1*H*-indol-4-yl)indolin-2-one (**4**): brown oil (75% yield); ^1^H NMR (500 MHz, CD_3_OD) *δ* 7.23 (d, *J* = 8.5 Hz, 1H), 7.20 (d, *J* = 8.5 Hz, 1H), 7.12 (s, 1H), 7.08 (s, 1H), 6.92 (d, *J* = 8.0 Hz, 2H), 6.66 (d, *J* = 8.5 Hz, 1H); ^13^C NMR (125 MHz, CD_3_OD) *δ* 181.2, 142.0, 136.4, 133.1, 128.8, 126.2, 125.8, 113.7, 113.0, 112.2, 94.6, 80.8; HRMS (ESI): calcd for C_16_H_10_ClN_2_O_3_^−^[M-1]^−^: 313.0385, found: 313.0380; [α]_D_^25^= −14.58 (c 0.43, MeOH). Enantiomeric excess (94%) was determined by chiral HPLC (Chiralpak AD-H), hexane:^i^PrOH = 80:20, 1.5 mL/min, major enantiomer t_R_ = 15.1 min, minor enantiomer t_R_ = 24.1 min.

(-)-(*R*)-5-chloro-3-hydroxy-3-(6-hydroxy-1*H*-indol-7-yl)indolin-2-one (**5**): brown oil (72% yield); ^1^H NMR (500 MHz, CD_3_OD) *δ* 7.30 (d, *J* = 8.5 Hz, 1H), 7.19 (dd, *J* = 2.0, 8.0 Hz, 1H), 7.15 (s, 1H), 6.99 (d, *J* = 2.0 Hz, 1H), 6.87 (d, *J* = 8.5 Hz, 2H), 6.49 (d, *J* = 8.5 Hz, 1H); ^13^C NMR (125 MHz, CD_3_OD) *δ*182.0, 150.2, 142.3, 137.2, 136.7, 129.9, 128.3, 125.4, 124.6, 124.0, 121.2, 111.9, 110.5, 109.7, 94.6, 79.6; HRMS (ESI): calcd for C_16_H_10_ClN_2_O_3_^−^[M-1]^−^: 313.0385, found: 313.0379; [α]_D_^25^ = −11.50 (c 0.56, MeOH). Enantiomeric excess (80%) was determined by chiral HPLC (Chiralpak AD-H), hexane:^i^PrOH = 80:20, 1.5 mL/min, major enantiomer t_R_ = 11.6 min, minor enantiomer t_R_ = 33.6 min.

(+)-(*R*)-5-chloro-3-hydroxy-3-(7-hydroxy-1*H*-indol-6-yl)indolin-2-one (**6**): brown oil (68% yield); ^1^H NMR (500 MHz, CD_3_OD) *δ* 7.29 (dd, *J* = 8.0, 2.0 Hz, 1H), 7.24 (d, *J* = 1.5 Hz, 1H), 7.20 (d, *J* = 3.0 Hz, 1H), 6.97 (d, *J* = 8.5 Hz, 1H), 6.93 (d, *J* = 8.5 Hz, 1H), 6.54 (d, *J* = 8.0 Hz, 1H), 6.35 (d, *J* = 3.0 Hz, 1H); ^13^C NMR (125 MHz, CD_3_OD) *δ* 182.0, 144.1, 141.8, 136.3, 131.6, 130.5, 128.5, 126.6, 126.2, 118.8, 115.6, 112.6, 112.5, 102.6, 81.0; HRMS (ESI): calcd for C_16_H_10_ClN_2_O_3_^−^ [M-1]^−^: 313.0385, found: 313.0386; [α]_D_^25^ = +9.07 (c 0.55, MeOH). Enantiomeric excess (60%) was determined by chiral HPLC (Chiralpak AD-H), hexane:^i^PrOH = 80:20, 1.5 mL/min, minor enantiomer t_R_ = 10.1 min, major enantiomer t_R_ = 12.8 min.

## Figures and Tables

**Table 1:**
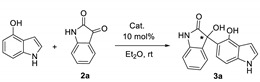
Asymmetric hydroxyalkylation of 4-hydroxyindole with isatin ^a^.

Entry	Catalyst ^a^	Yield (%) ^b^	%ee ^c^	Configuration ^d^
1	1a	70	68	R
2	1b	88	79	R
3	1c	75	72	S
4	1d	85	76	S
5	1e	-	-	-
6	1f	72	55	S
7	1g	65	35	S
8	1h	66	46	S

^a^ Reaction conditions: isatin (0.1 mmol), 4-hydroxyindole (0.1 mmol), catalyst (0.01 mmol), and Et_2_O (1.5 mL) at rt. ^b^ Isolated yield. ^c^Determined by HPLC analysis (Chiralpak AD-H). ^d^Absolute configuration was determined according to the literature [52].

**Table 2:** Screening of the reaction conditions for the asymmetric hydroxyalkylation ^a^.

Entry.	Solvent	Temperature	Catalyst. Amount (% mmol)	Yield (%) ^b^	%ee ^c^
1	Et_2_O	rt	10	88	79
2	DCM	rt	10	69	32
3	toluene	rt	10	70	28
4	THF	rt	10	76	69
5 ^d^	Et_2_O	rt	10	79	74
6	Et_2_O	rt	5	79	77
7	Et_2_O	rt	20	91	76
8	Et_2_O	0 °C	10	85	83
9	Et_2_O	0 °C	20	83	80
10	Et_2_O	−20 °C	10	72	68
11	Et_2_O	−40 °C	10	68	73

^a^ Reaction conditions: isatin (0.1 mmol), 4-hydroxyindole (0.1 mmol), catalyst 1b in solvent (1.5 mL). ^b^ Isolated yield. ^c^ Determined by HPLC analysis (Chiralpak AD-H). ^d^ Reaction performed in Et_2_O (3.0 mL).

**Table 3:**
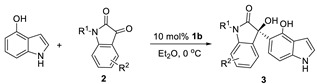
Scope of the enantioselective hydroxyalkylation between 4-hydroxyindole and isatins ^a^.

Entry	R^1^, R^2^	Product	Yield (%) ^b^	%ee ^c^
1	R^1^ = H, R^2^ = H, 2a	3a	85(70) ^d^	83(85) ^d^
2	R^1^ = H, R^2^ = 4-Br, 2b	3b	65	50
3	R^1^ = H, R^2^ = 5-Me, 2c	3c	85	85
4	R^1^ = H, R^2^ = 5-OMe, 2d	3d	78	82
5	R^1^ = H, R^2^ = 5-Cl, 2e	3e	92	94
6	R^1^ = H, R^2^ = 5-Br, 2f	3f	80(69) ^d^	90(84) ^d^
7	R^1^ = H, R^2^ = 6-Br, 2g	3g	83	71
8	R^1^ = H, R^2^ = 7-F, 2h	3h	90	92
9	R^1^ = H, R^2^ = 7-Cl, 2i	3i	81	84
10	R^1^ = Me, R^2^ = H, 2j	3j	82(77) ^d^	78(80) ^d^
11	R^1^ = Bn, R^2^ = H, 2k	3k	87(91) ^d^	74(90) ^d^

^a^ Reaction conditions: isatin (0.1 mmol), 4-hydroxyindole (0.1 mmol), and catalyst (0.01 mmol) in Et_2_O (1.5 mL). ^b^Isolated yield. ^c^ Determined by HPLC analysis (Chiralpak AD-H). ^d^ The data in parentheses are the ee values and yield in [52].

## Supplementary Materials

Copies of ^1^H and ^13^C-NMR spectra and HPLC trace of products are available online at https://www.mdpi.com/1420-3049/24/21/3944/s1. Figures S1–S25: NMR spectra of compounds 3a–3k and 4–6; Figures S26–29: HPLC trace of enantiomeric 3a in different solvent. S30–S57: HPLC trace of compounds 3a–3k and 4–6.
