# Supplementary material for: Urea Derivative Catalyzed Enantioselective Hydroxyalkylation of Hydroxyindoles with Isatins
Source: Molecules. 2019 Oct 31;24(21):3944. doi: 10.3390/molecules24213944 (PMC6864875; doi:10.3390/molecules24213944)
Supplement: Supplementary file 1 [file molecules-24-03944-s001.pdf]

Supporting Information

Urea Derivative Catalyzed Enantioselective Hydroxyalkylation of Hydroxyindoles with Isatins

Hao Wu, Liming Wang, Junwei Zhang, Ying Jin*

(*Department of Pharmacy, Jilin Medical University, Jilin, Jilin 132013, China)*

^{1}H NMR and ^{13}C NMR spectra……………………..….………........…S2-S14

HPLC trace of **3a** in different solvent at rt………………...……….....… S15-S17

HPLC trace of **3a-k** and **4, 5, 6** at 0 °C………….……..……...………… S18-S30

^{1}H NMR and ^{13}C NMR spectra

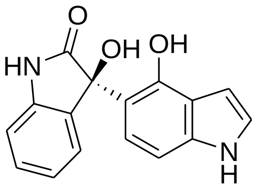

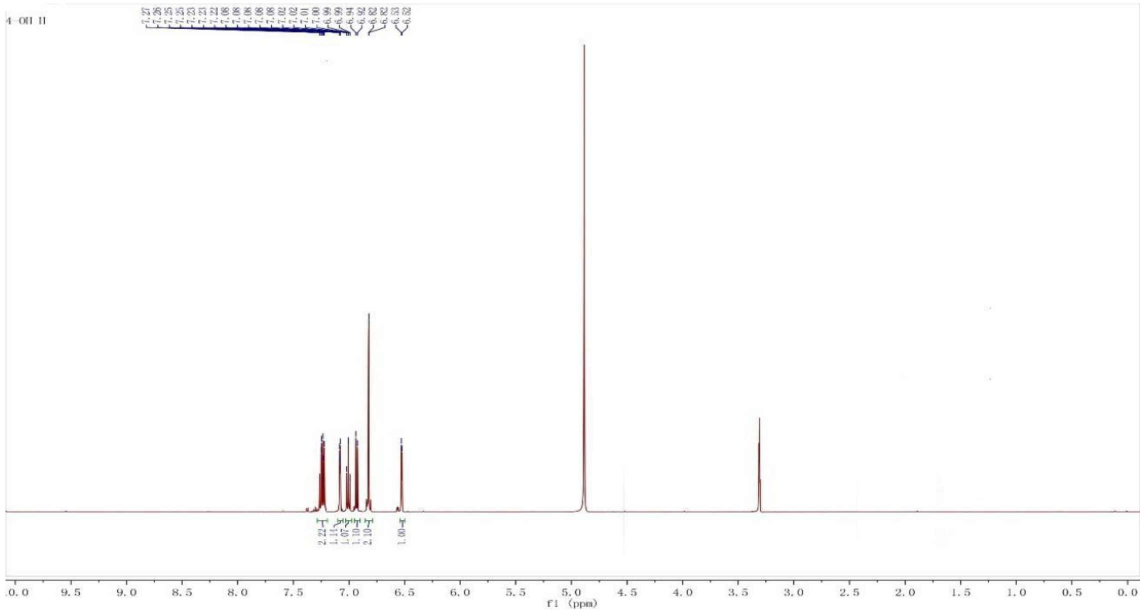

Figure S1. ^{1}H NMR spectrum of **3a**

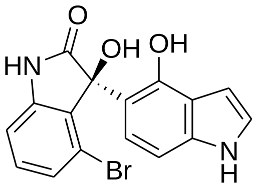

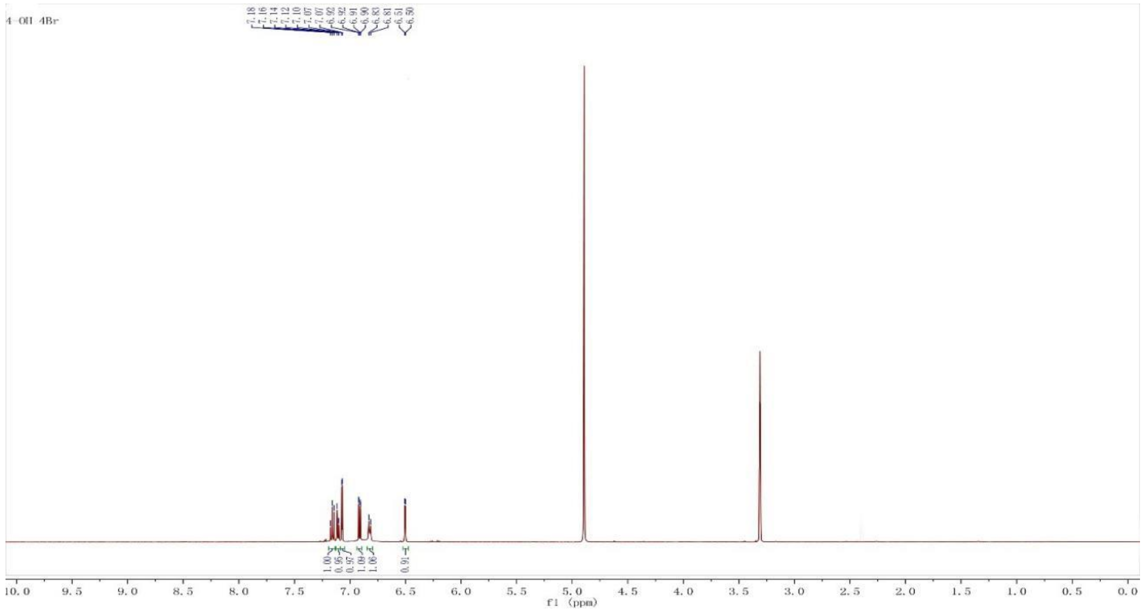

Figure S2. ^{1}H NMR spectrum of **3b**

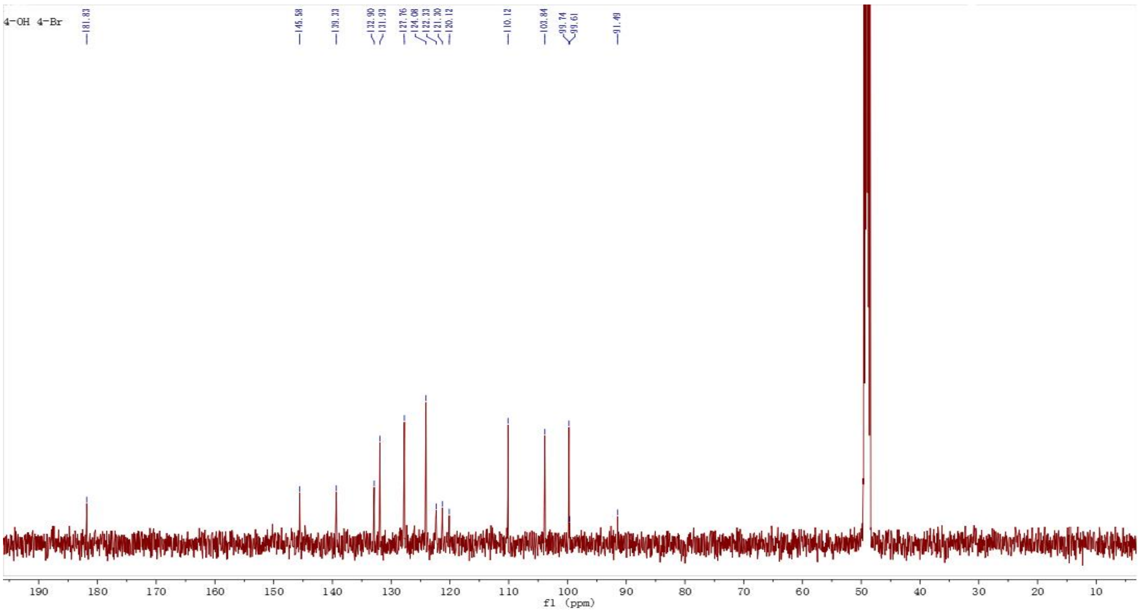

Figure S3. ^{13}C NMR spectrum of **3b**

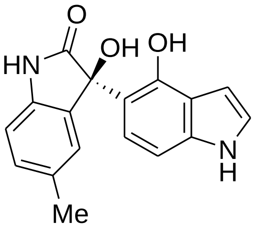

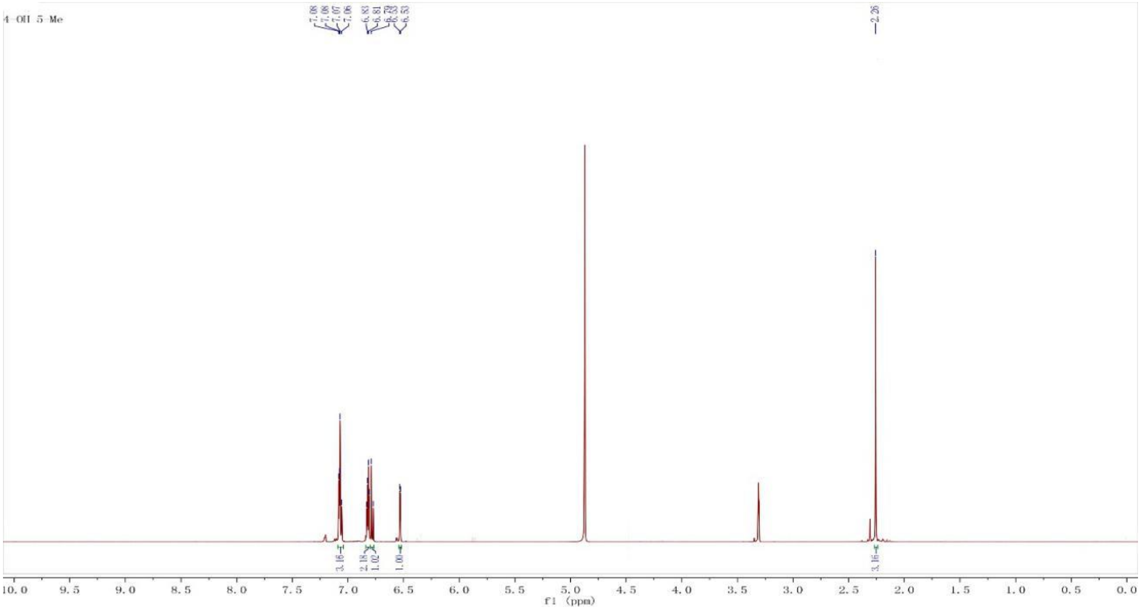

Figure S4. ^{1}H NMR spectrum of **3c**

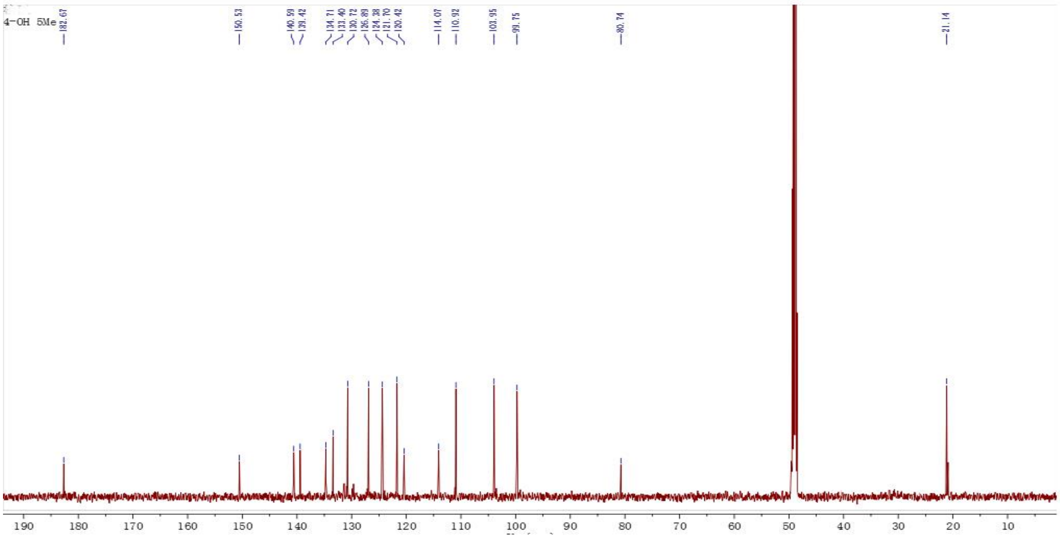

Figure S5. ^{13}C NMR spectrum of **3c**

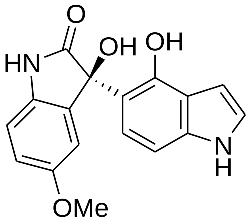

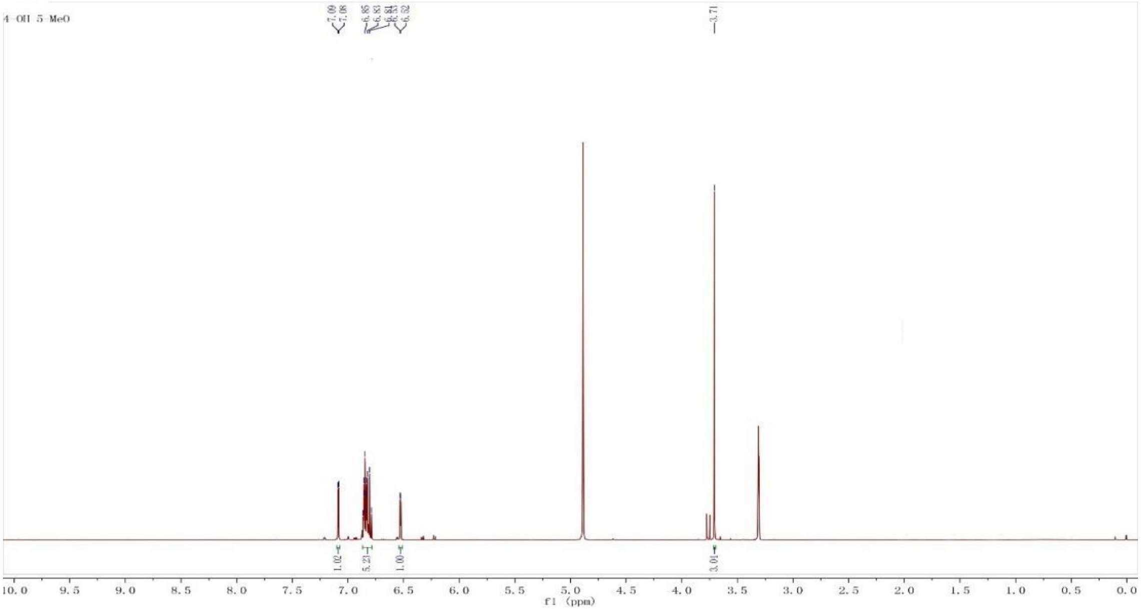

Figure S6. ^{1}H NMR spectrum of **3d**

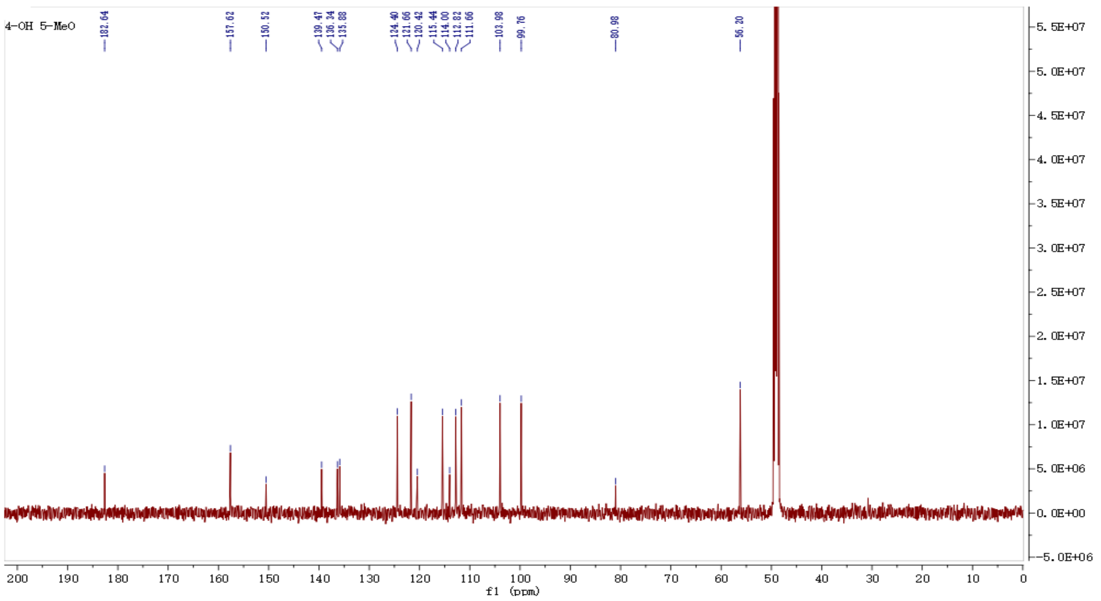

Figure S7. ^{13}C NMR spectrum of **3d**

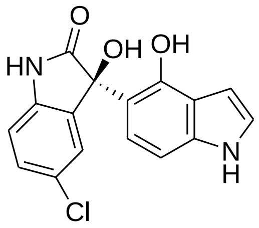

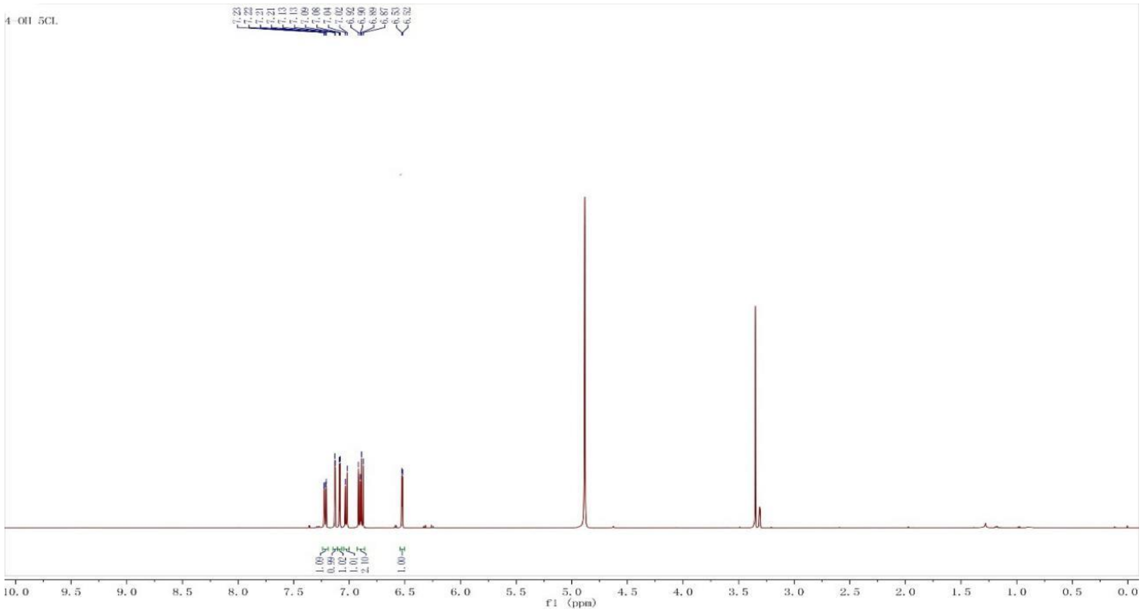

Figure S8. ^{1}H NMR spectrum of **3e**

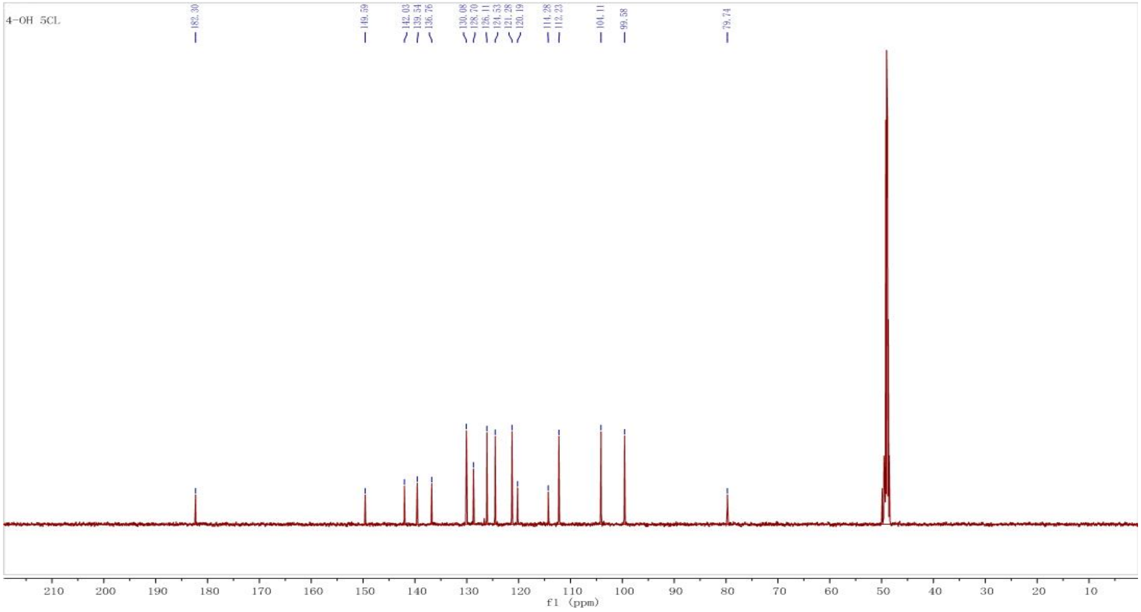

Figure S9. ^{13}C NMR spectrum of **3e**

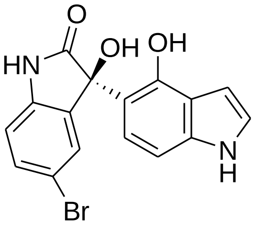

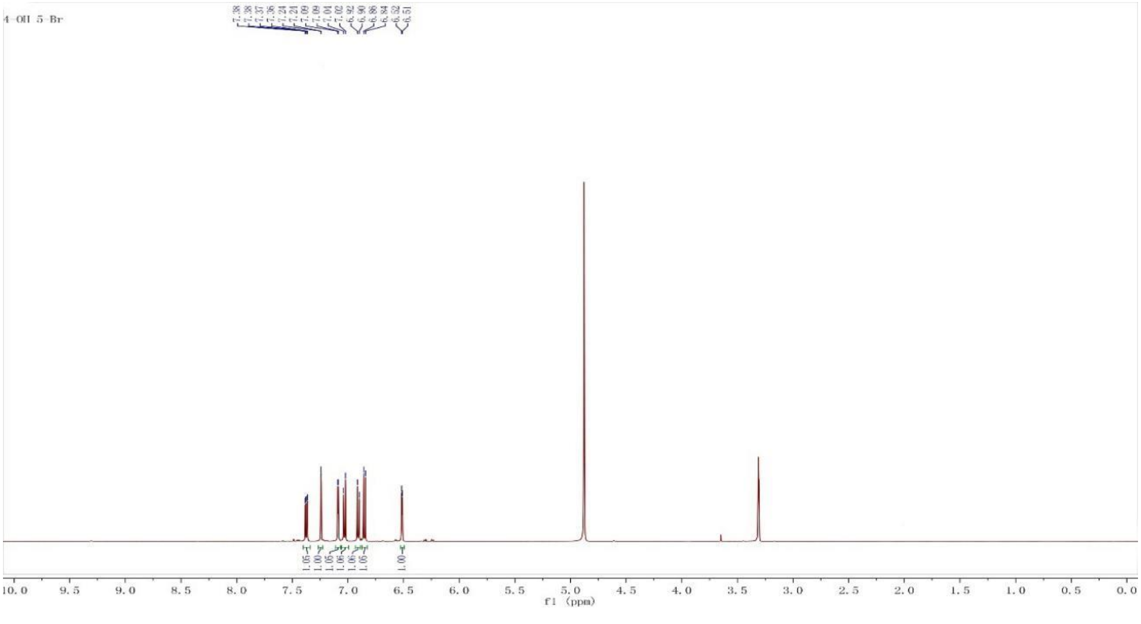

Figure S10 ^{1}H NMR spectrum of **3f**

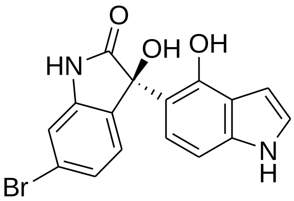

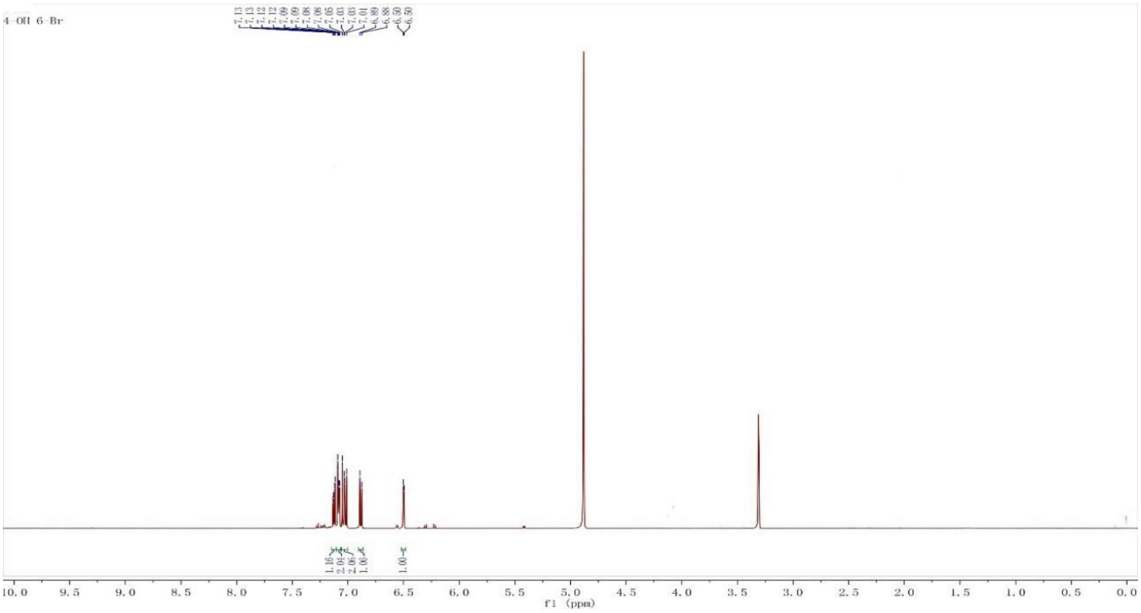

Figure S11. ^{1}H NMR spectrum of **3g**

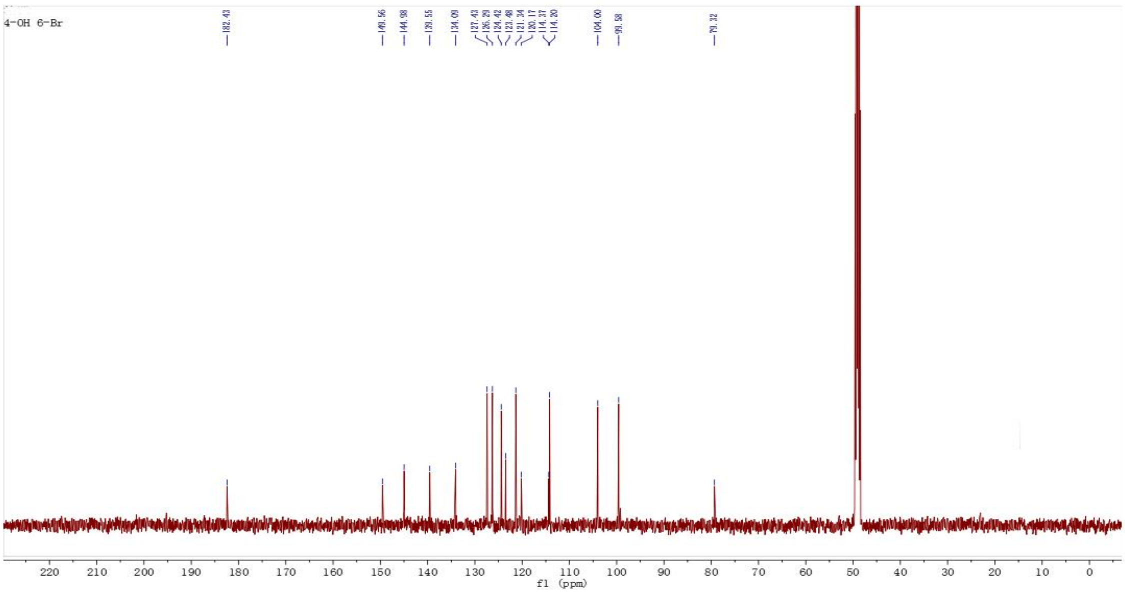

Figure S12. ^{13}C NMR spectrum of **3g**

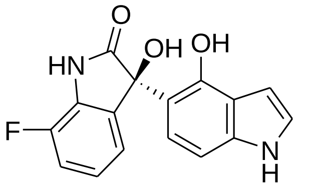

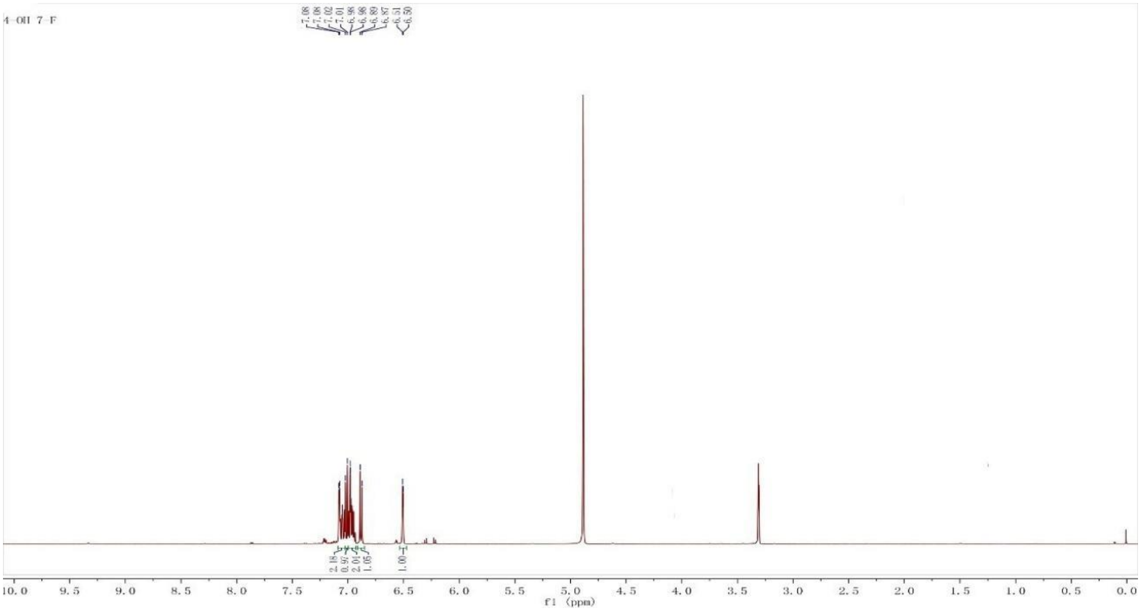

Figure S13. ^{1}H NMR spectrum of **3h**

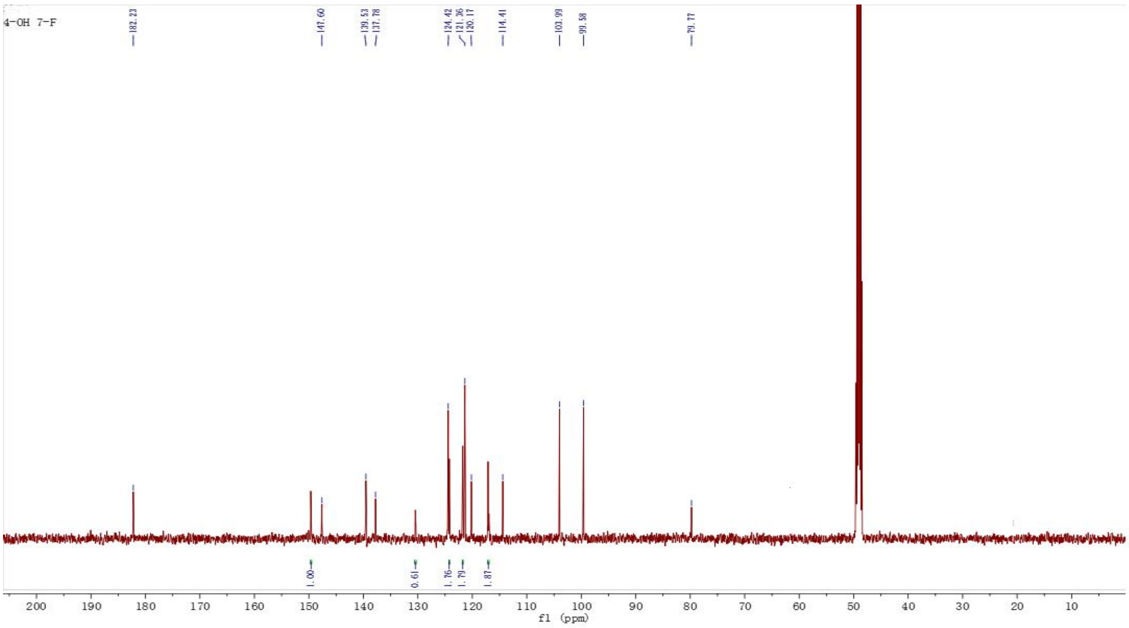

Figure S14. ^{13}C NMR spectrum of **3h**

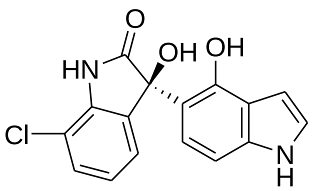

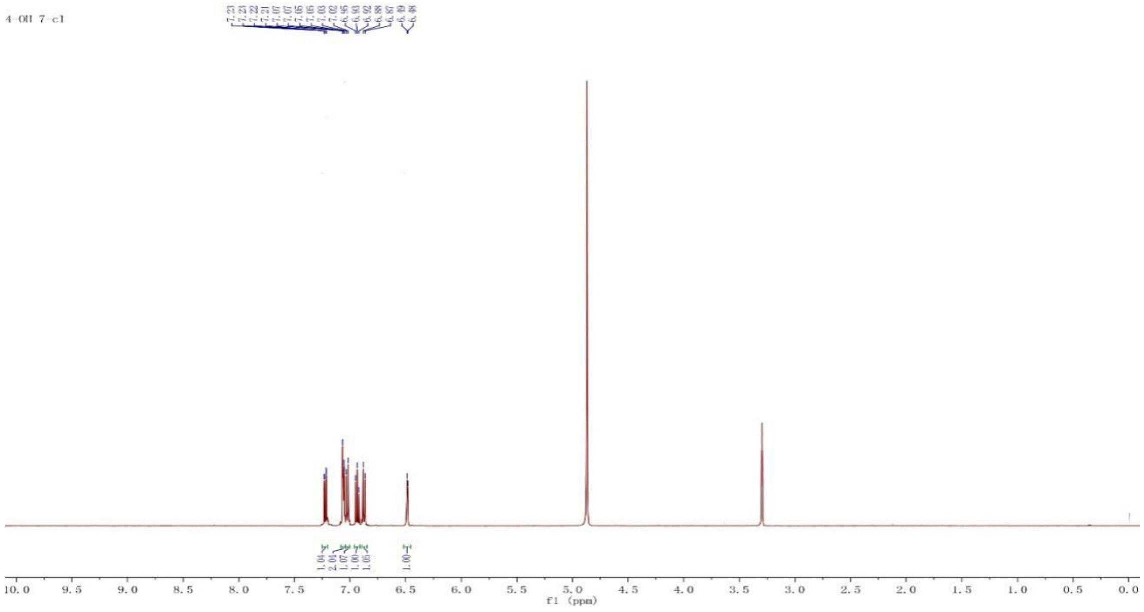

Figure S15. ^{1}H NMR spectrum of **3i**

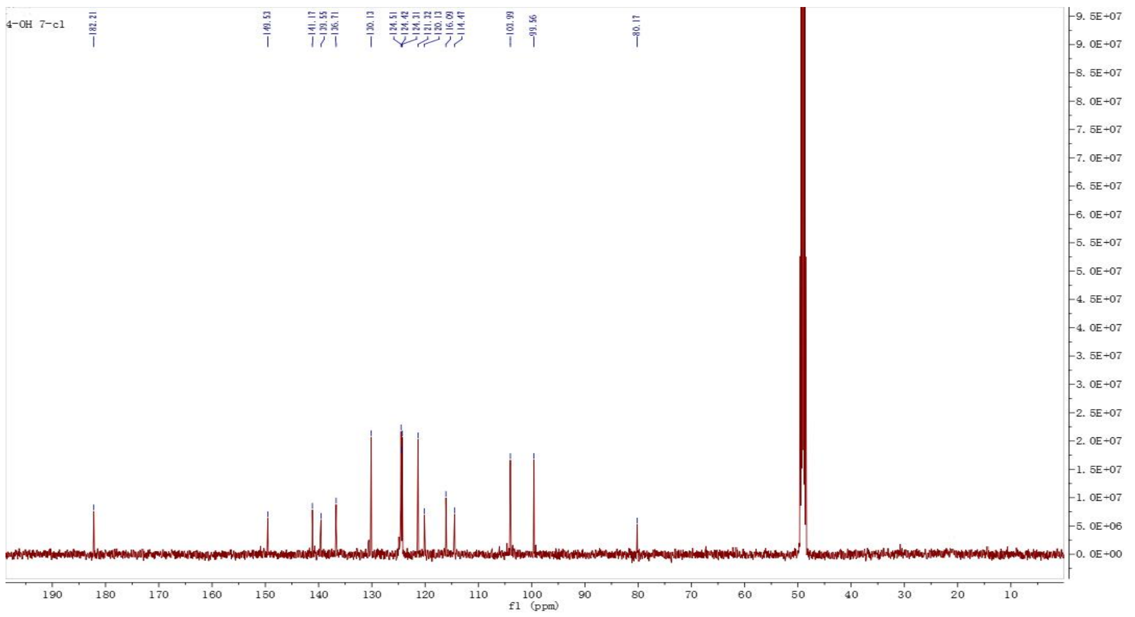

Figure S16. ^{13}C NMR spectrum of **3i**

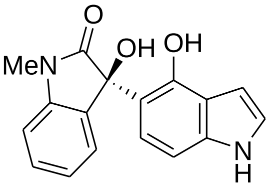

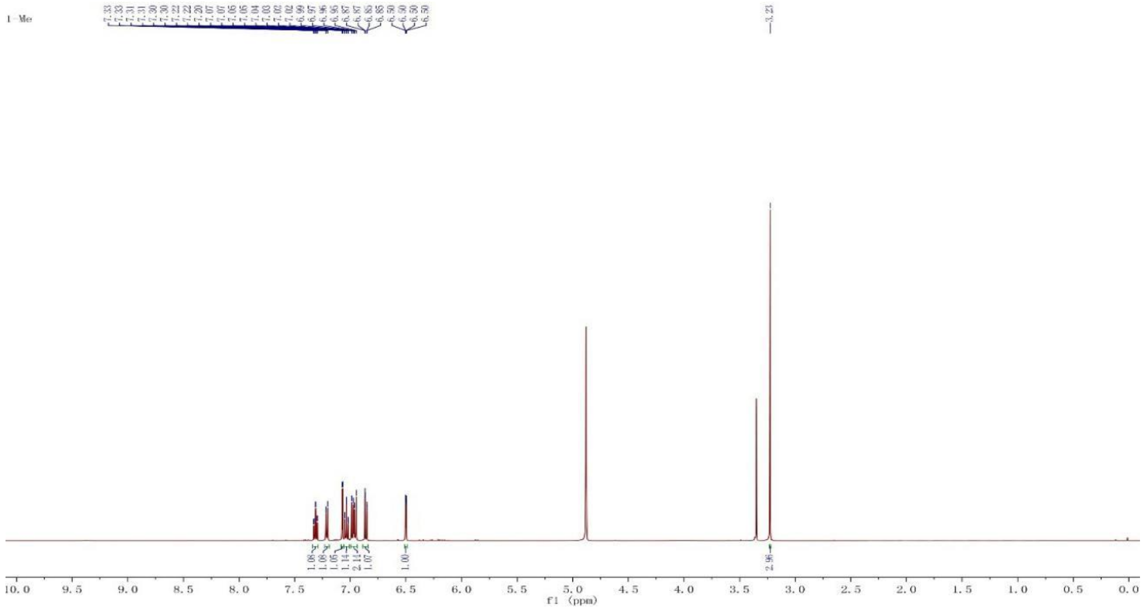

Figure S17. ^{1}H NMR spectrum of **3j**

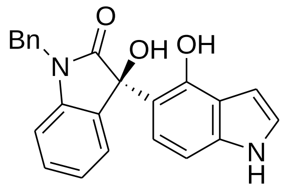

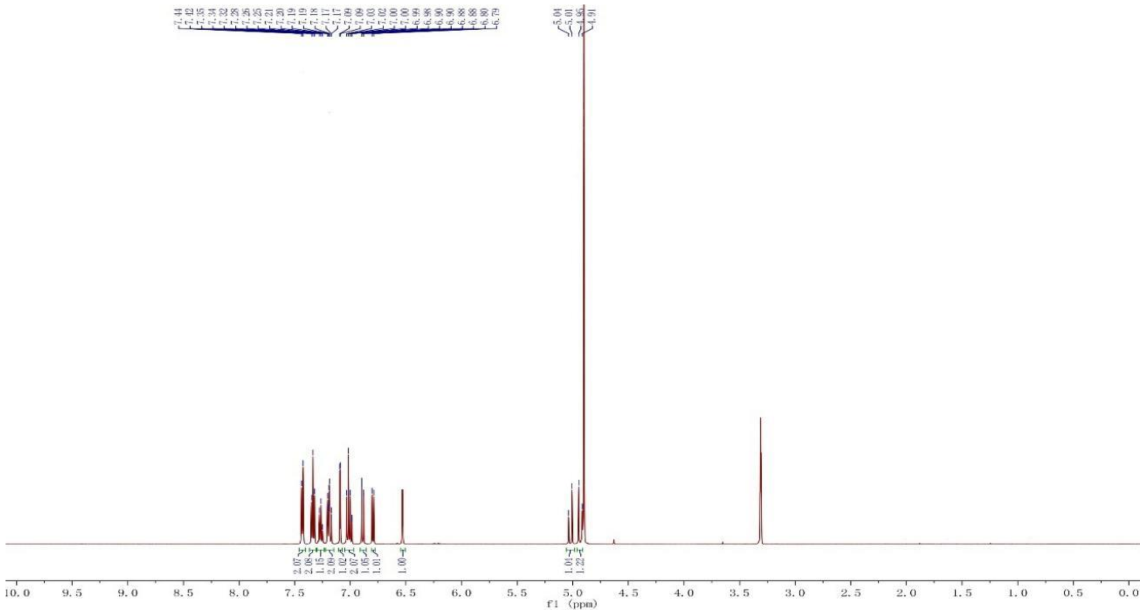

Figure S18. ^{1}H NMR spectrum of **3k**

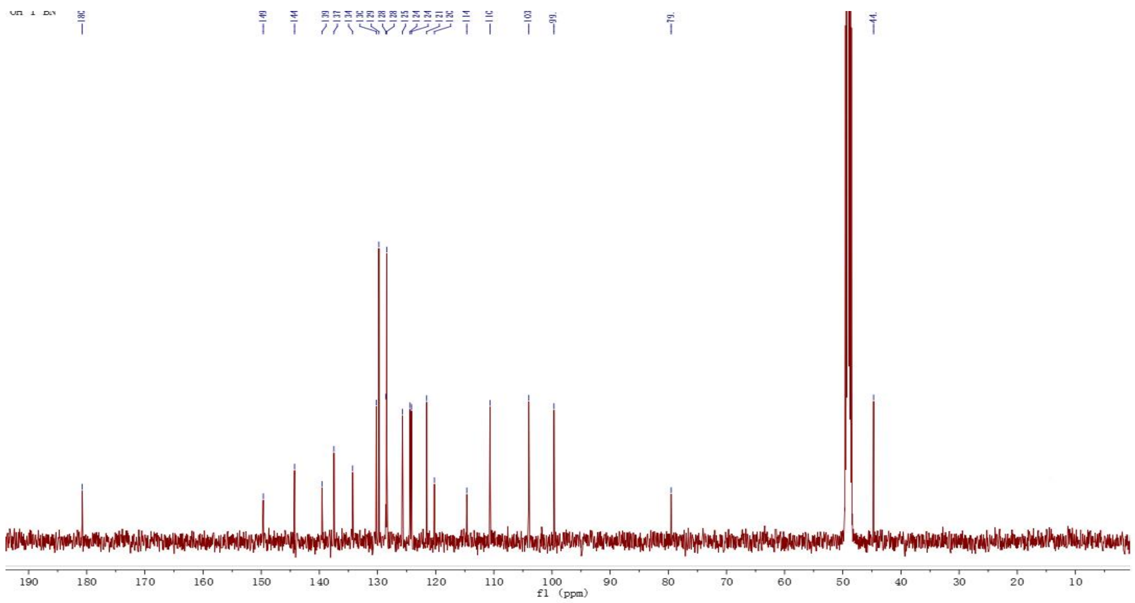

Figure S19. ^{13}C NMR spectrum of **3k**

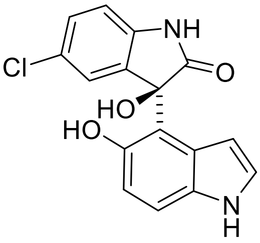

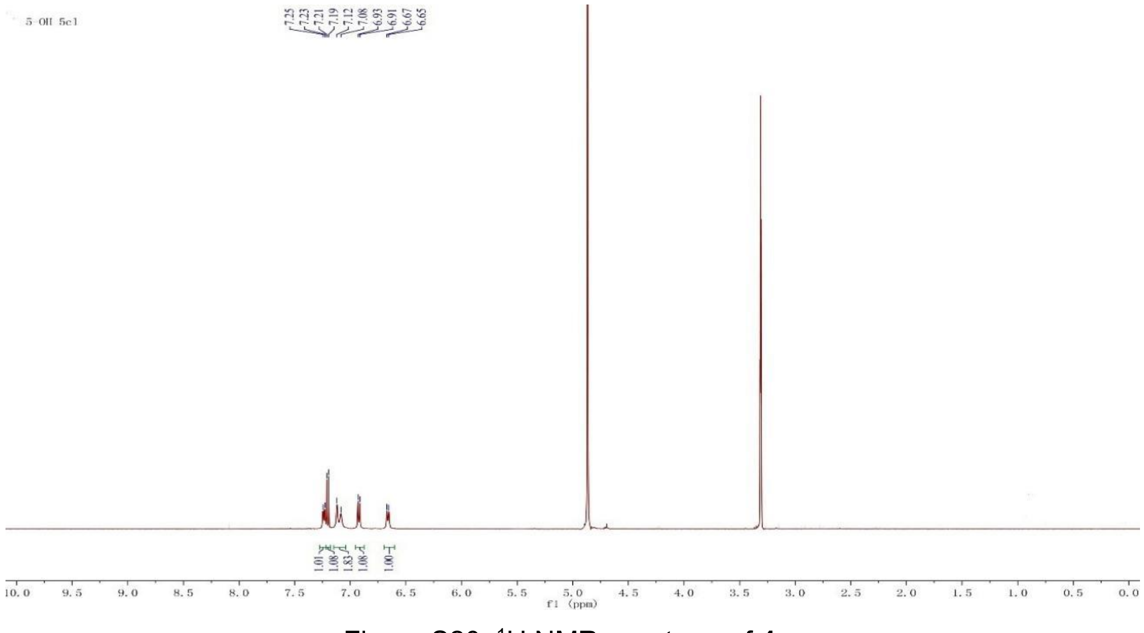

Figure S20. ^{1}H NMR spectrum of **4**

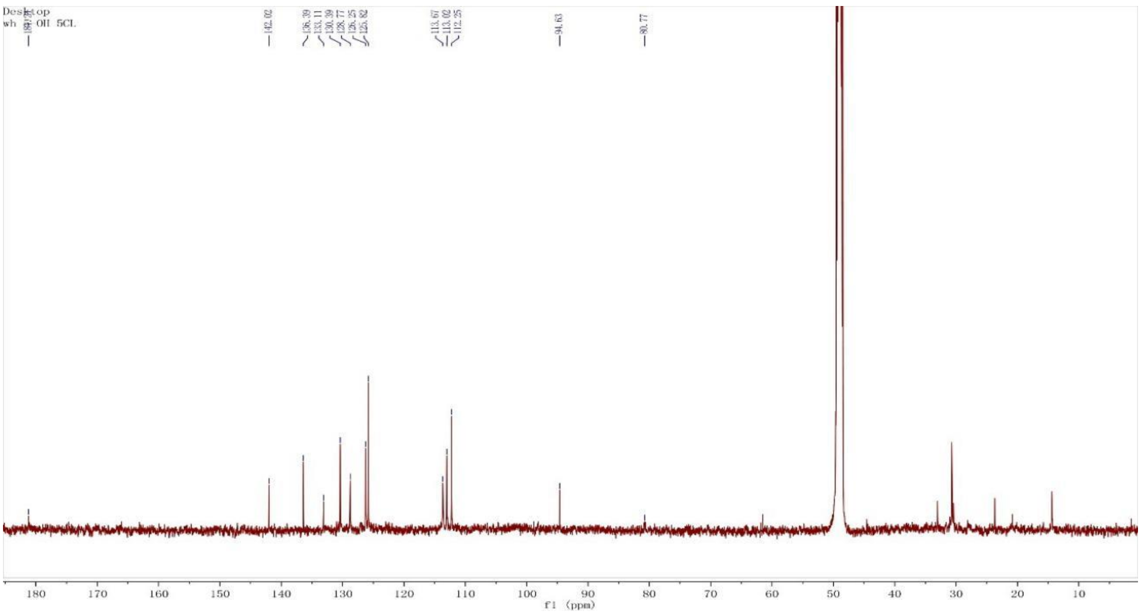

Figure S21. ^{13}C NMR spectrum of **4**

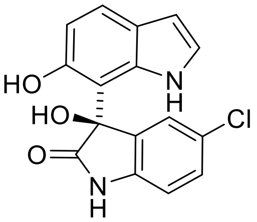

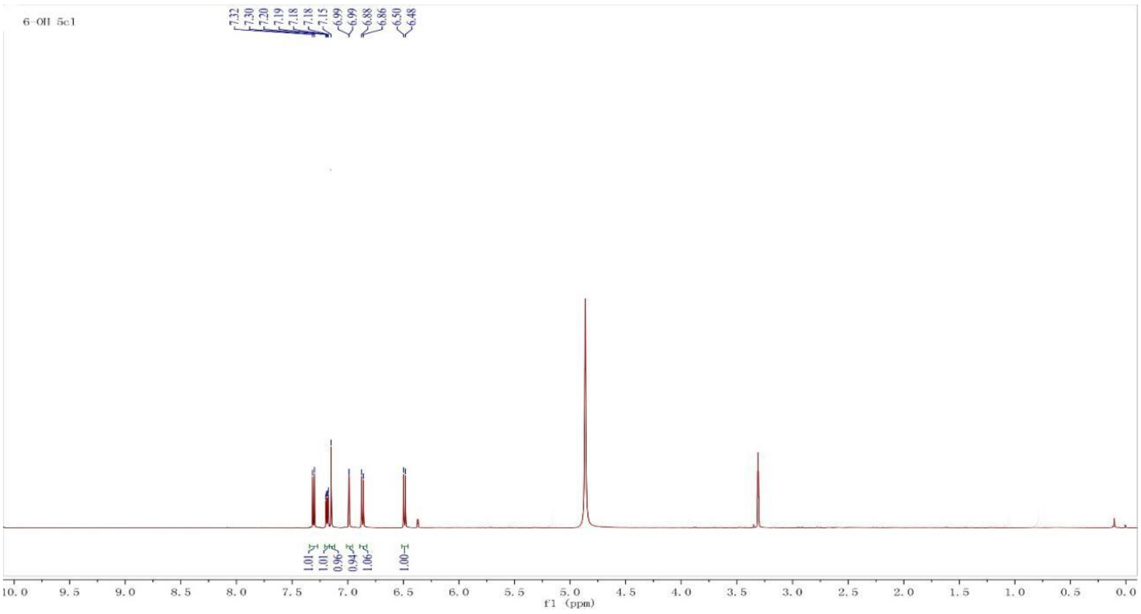

Figure S22. ^{1}H NMR spectrum of **5**

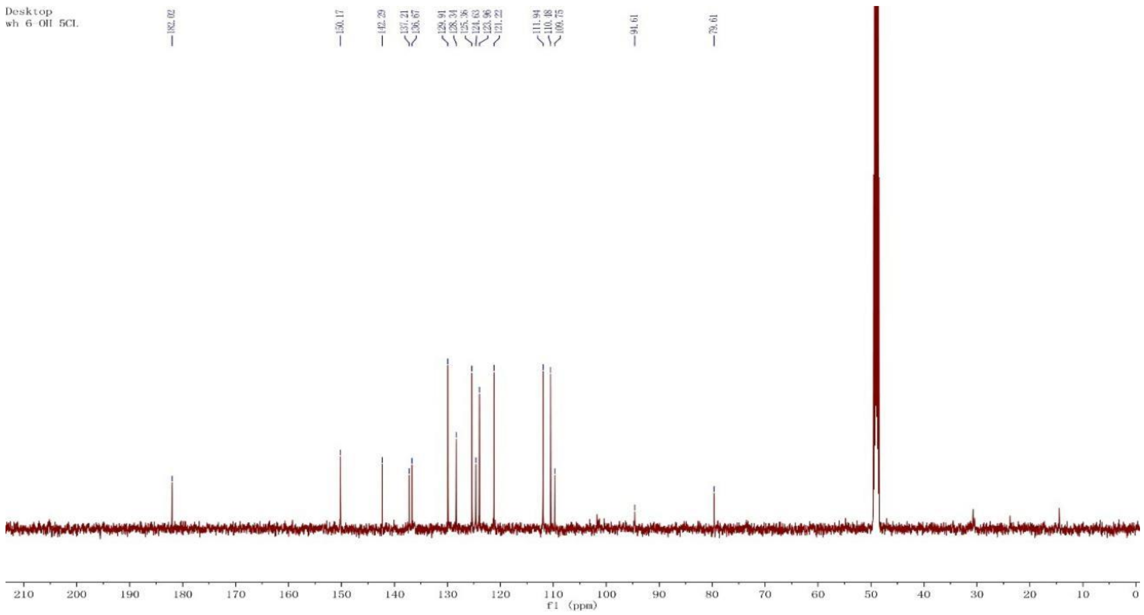

Figure S23. ^{13}C NMR spectrum of **5**

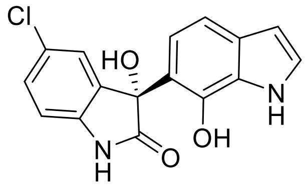

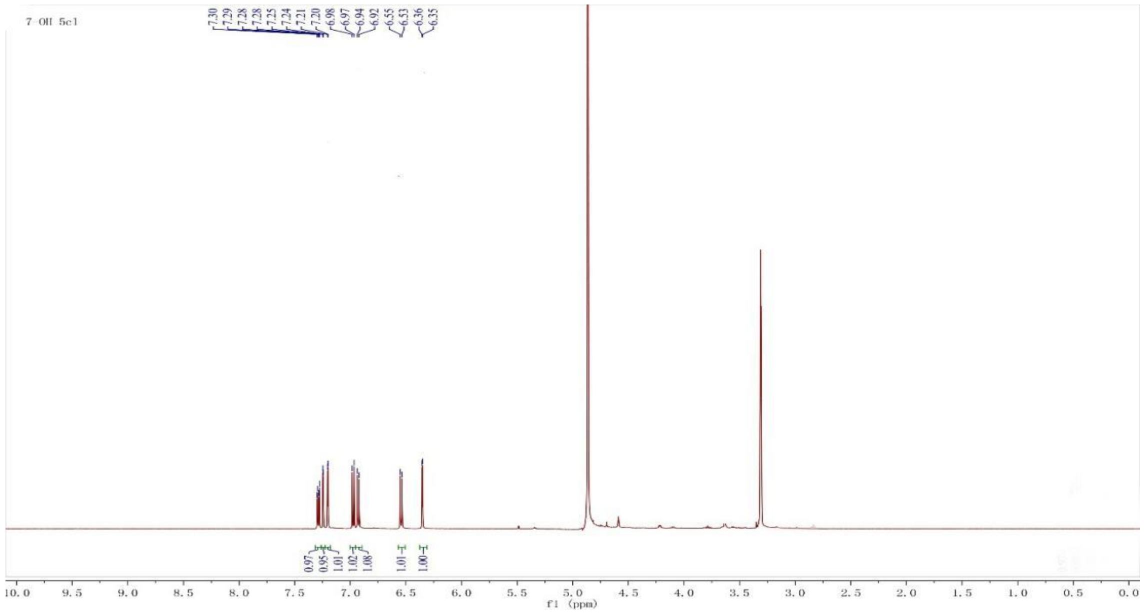

Figure S24. ^{1}H NMR spectrum of **6**

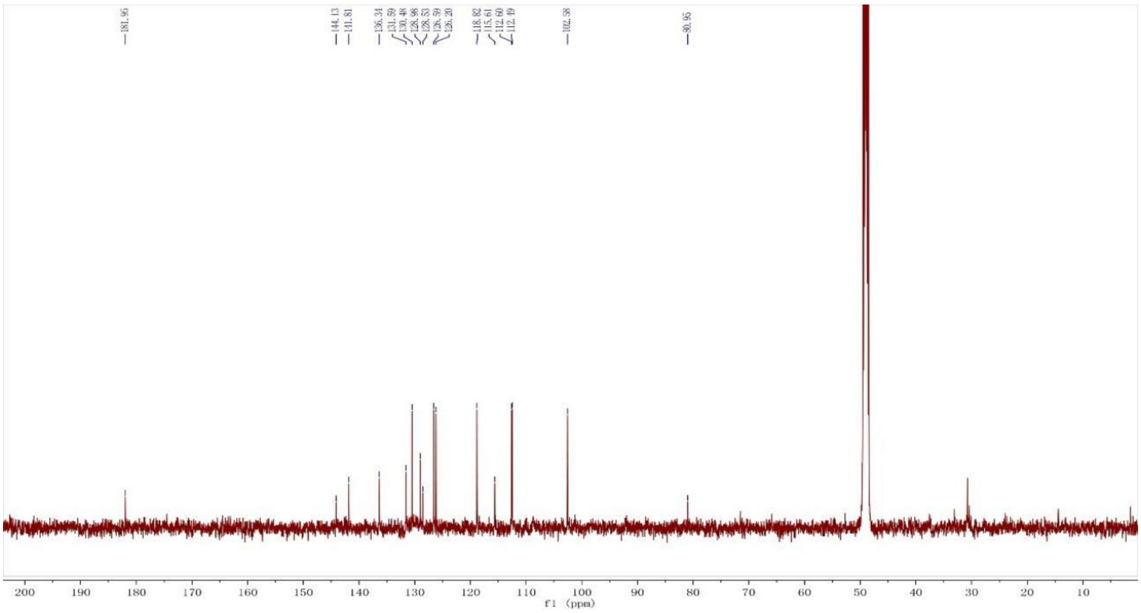

Figure S25. ^{13}C NMR spectrum of **6**

HPLC trace

HPLC trace of **3a** in different solvent at rt:

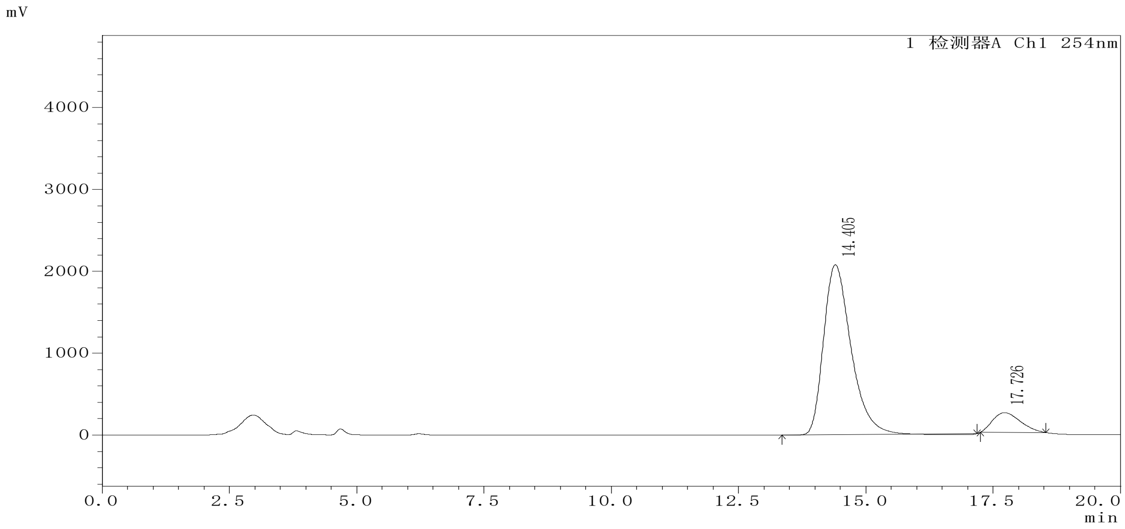

Figure S26. HPLC trace of enantiomeric **3a** with **Et_2O** as solvent

	Retention Time	Area	Area percent
1	14.405	76971273	89.372
2	17.726	9153706	10.628
Total		86124978	100.000

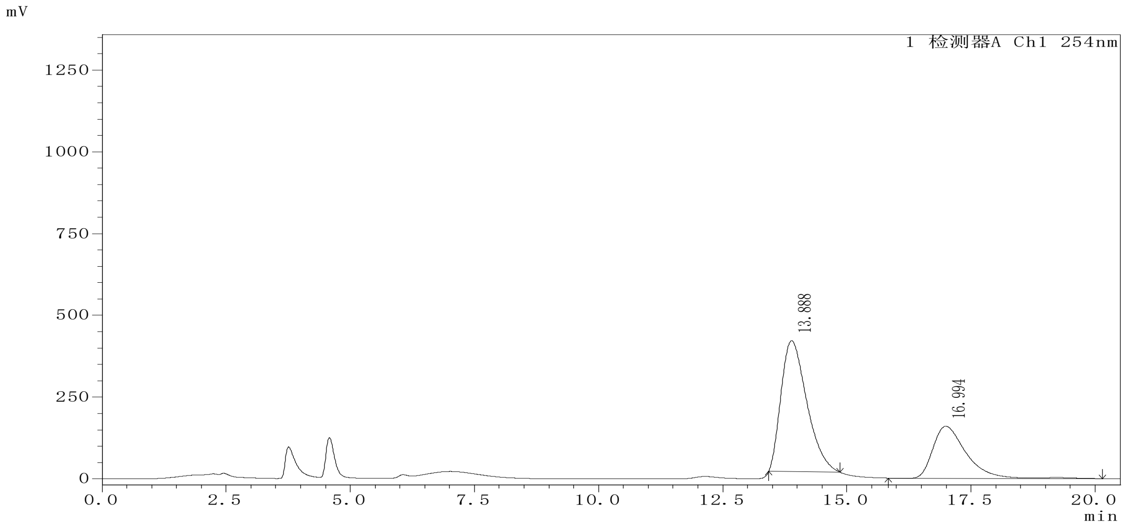

Figure S27. HPLC trace of enantiomeric **3a** with **DCM** as solvent

	Retention Time	Area	Area percent
1	13.888	14547047	66.165
2	16.994	7438845	33.835
Total		21985893	100.000

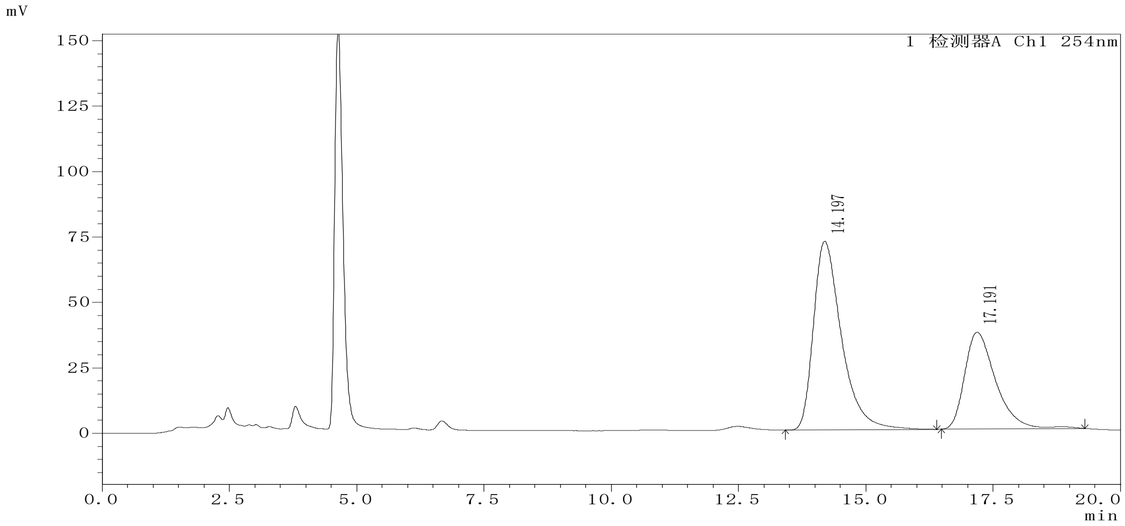

Figure S28. HPLC trace of enantiomeric **3a** with **toluene** as solvent

	Retention Time	Area	Area percent
1	14.197	2767222	63.789
2	17.191	1570894	36.211
Total		4338116	100.000

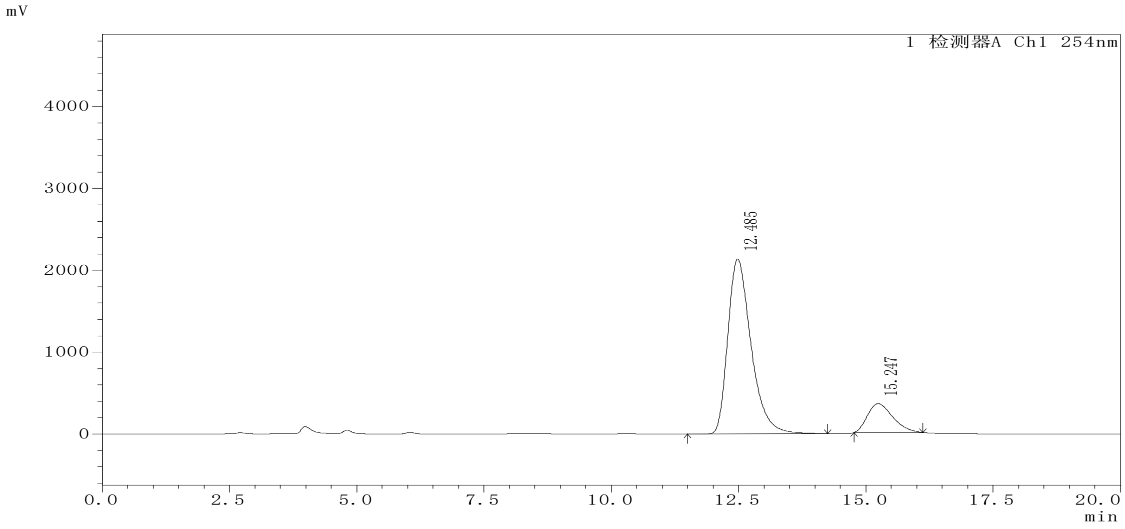

Figure S29. HPLC trace of enantiomeric **3a** with **THF** as solvent

	Retention Time	Area	Area percent
1	12.485	67726383	84.658
2	15.247	12273243	15.342
Total		79999626	100.000

HPLC trace of **3a-k** and **4,5,6** at 0 °C

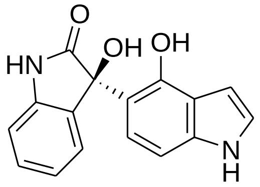

3a

3-Hydroxy-3-(4-hydroxy-1*H*-indol-5-yl)indolin-2-one

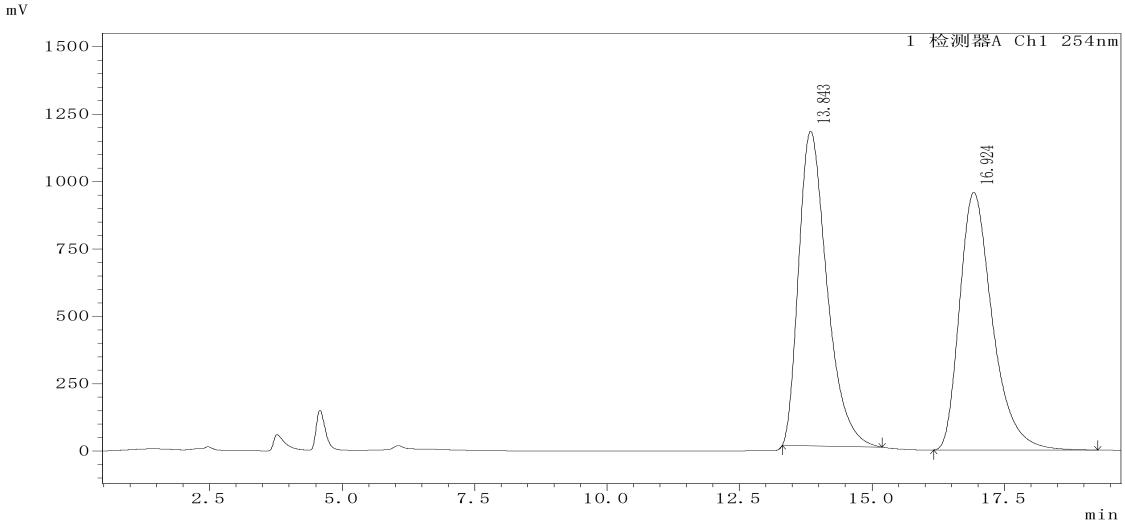

Figure S30. HPLC trace of racemic **3a**

	Retention Time	Area	Area percent
1	13.843	43424937	51.239
2	16.924	41324222	48.761
Total		84749159	100.000

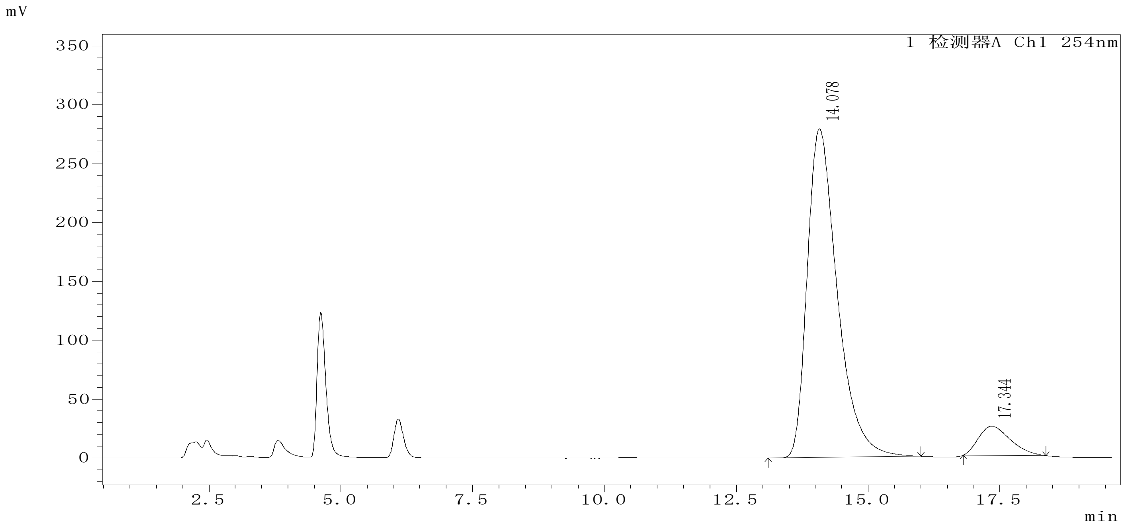

Figure S31. HPLC trace of enantiomeric **3a**

	Retention Time	Area	Area percent
1	14.078	11057977	91.348
2	17.344	1047397	8.652
Total		12105374	100.000

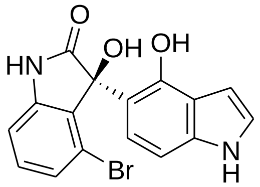

3b

4-bromo-3-hydroxy-3-(4-hydroxy-1*H*-indol-5-yl)indolin-2-one

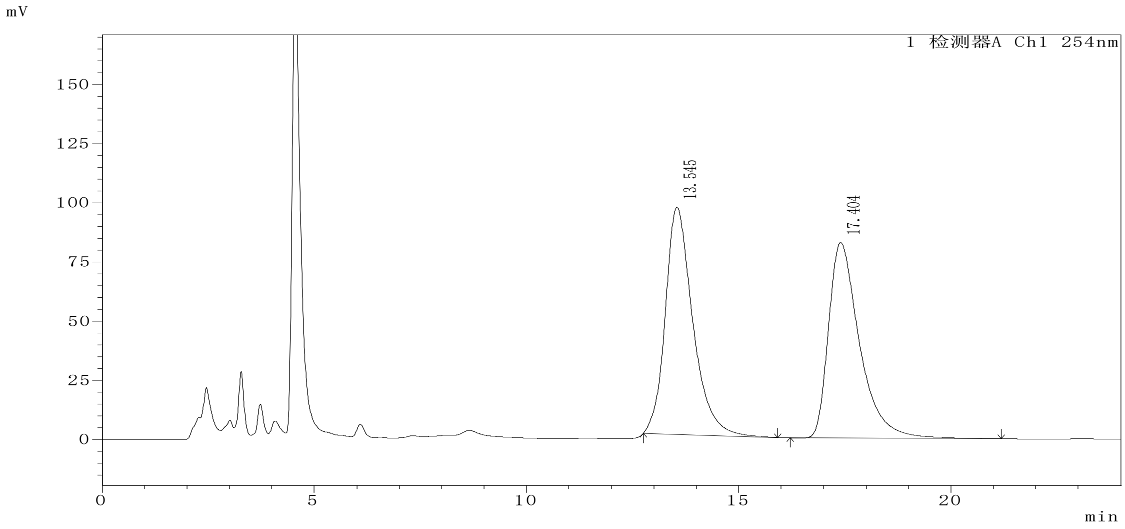

Figure S32. HPLC trace of racemic **3b**

	Retention Time	Area	Area percent
1	13.545	4338091	50.833
2	17.404	4195873	49.167
Total		8533964	100.000

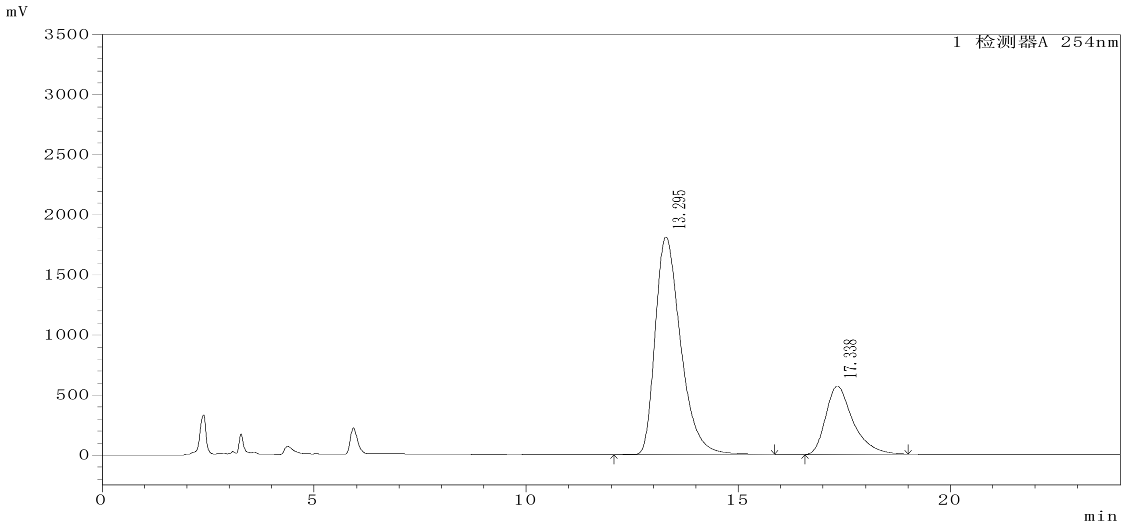

Figure S33. HPLC trace of enantiomeric **3b**

	Retention Time	Area	Area percent
1	13.295	75609794	74.837
2	17.338	25423375	25.163
Total		101033169	100.000

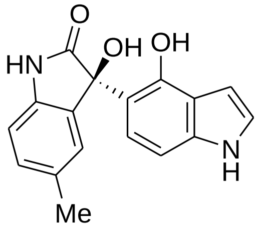

3c

5-methyl-3-hydroxy-3-(4-hydroxy-1*H*-indol-5-yl)indolin-2-one

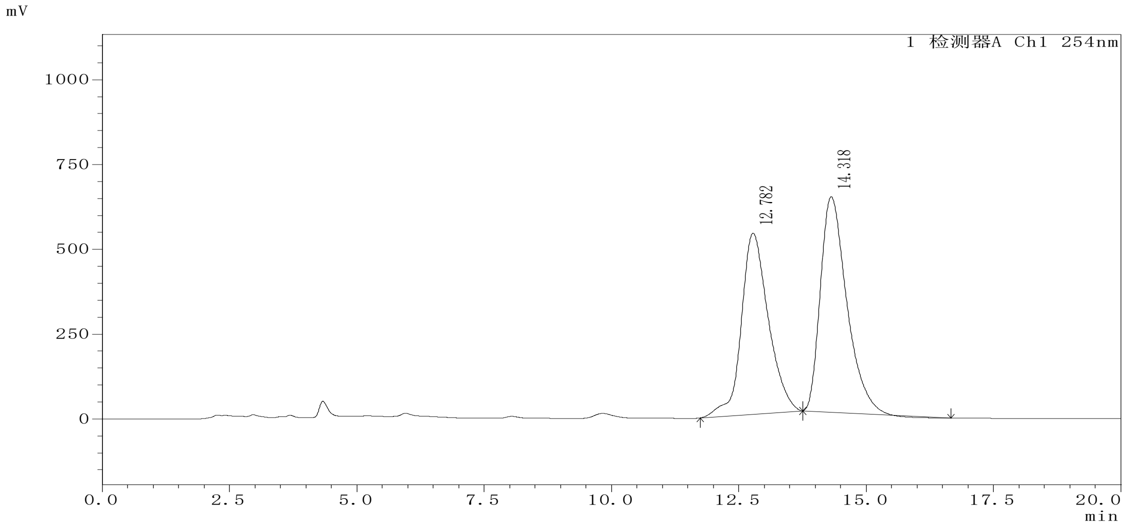

Figure S34. HPLC trace of racemic **3c**

	Retention Time	Area	Area percent
1	12.782	19754440	46.363
2	14.318	22853934	53.637

Total		42608373	100.000

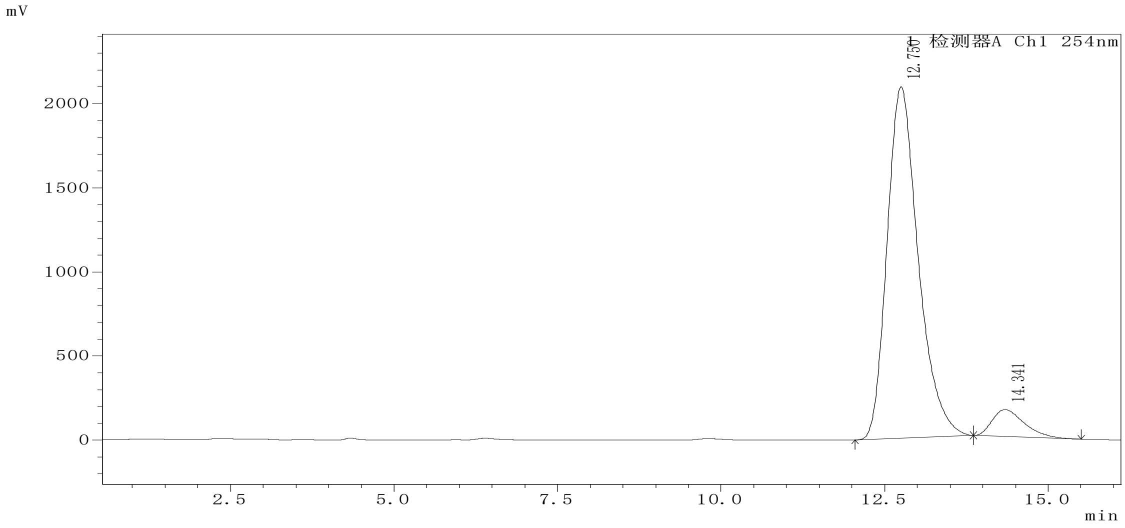

Figure S35. HPLC trace of enantiomeric **3c**

	Retention Time	Area	Area percent
1	12.750	69355750	92.630
2	14.341	5517924	7.370
Total		74873674	100.000

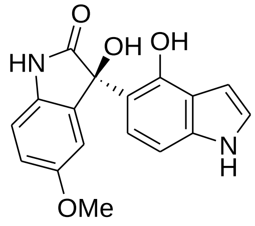

3d

5-methoxyl-3-hydroxy-3-(4-hydroxy-1*H*-indol-5-yl)indolin-2-one

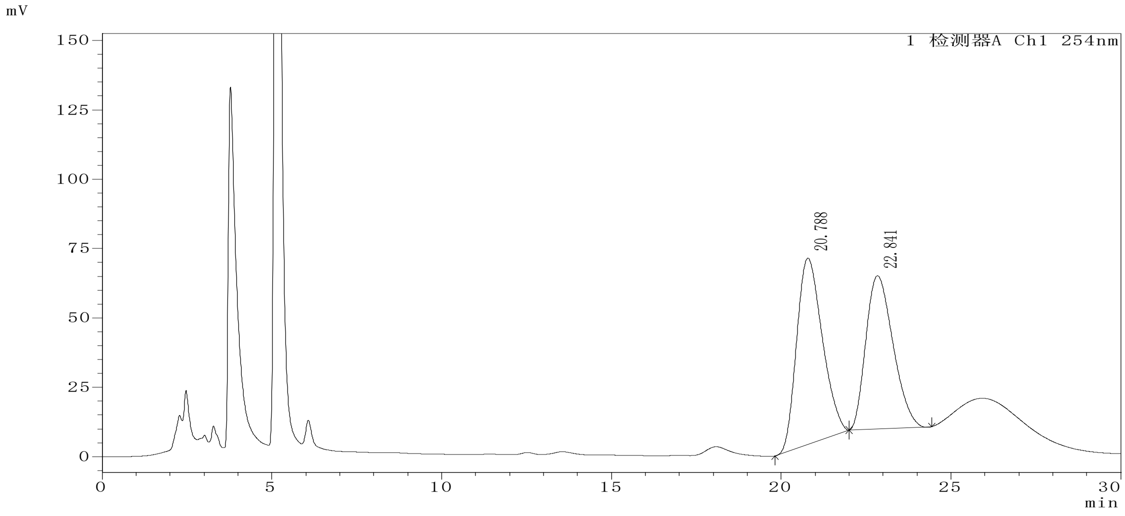

Figure S36. HPLC trace of racemic **3d**

	Retention Time	Area	Area percent
1	20.788	3533671	53.451
2	22.841	3077328	46.549

Total		6610999	100.000

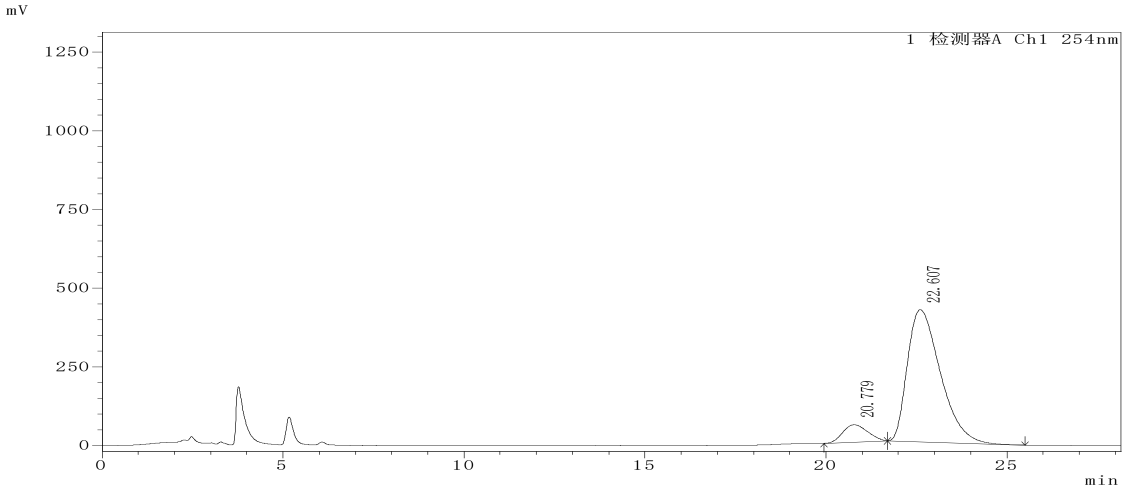

Figure S37. HPLC trace of enantiomeric **3d**

	Retention Time	Area	Area percent
1	20.779	2633961	9.015
2	22.607	26584869	90.985
Total		29218830	100.000

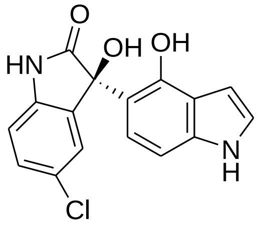

3e

5-chloro-3-hydroxy-3-(4-hydroxy-1*H*-indol-5-yl)indolin-2-one

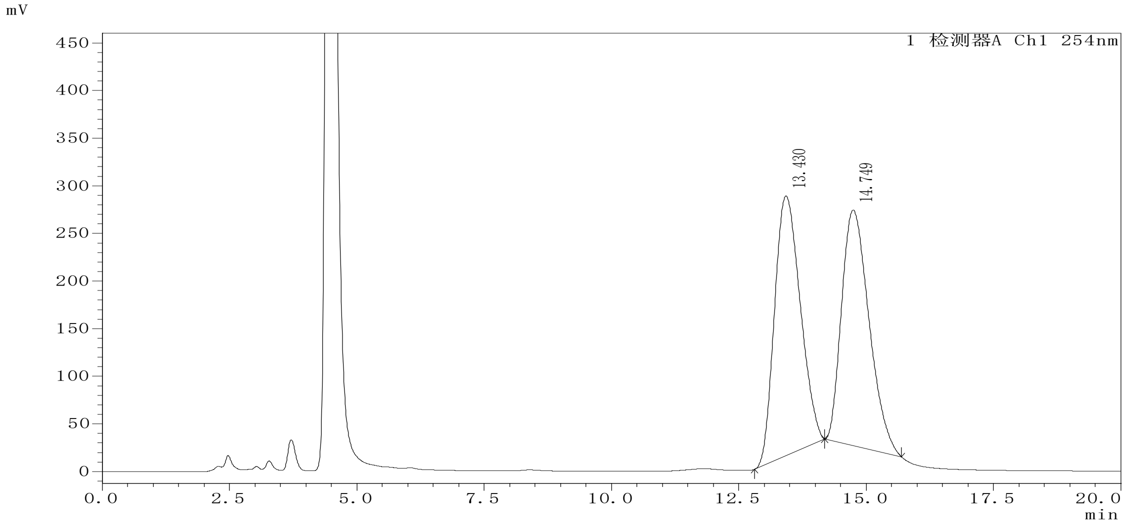

Figure S38. HPLC trace of racemic **3e**

	Retention Time	Area	Area percent
1	13.430	9604851	50.825
2	14.749	9293112	49.175

Total		18897963	100.000

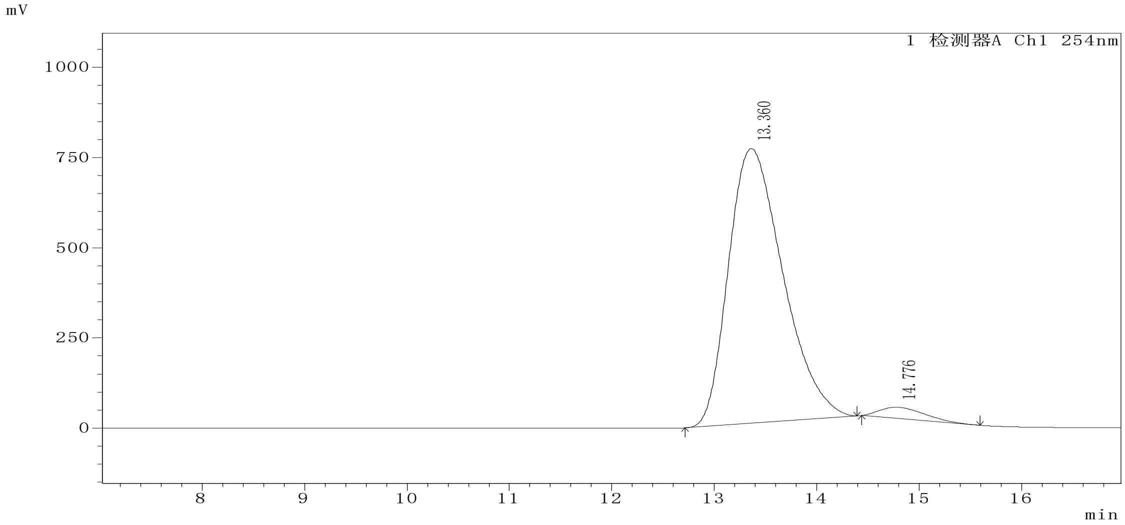

Figure S39. HPLC trace of enantiomeric **3e**

	Retention Time	Area	Area percent
1	13.360	28204931	96.722
2	14.776	955849	3.278
Total		29160781	100.000

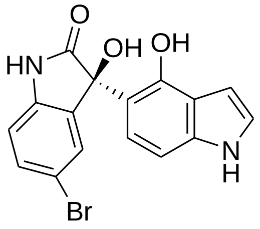

3f

5-bromo-3-hydroxy-3-(4-hydroxy-1*H*-indol-5-yl)indolin-2-one

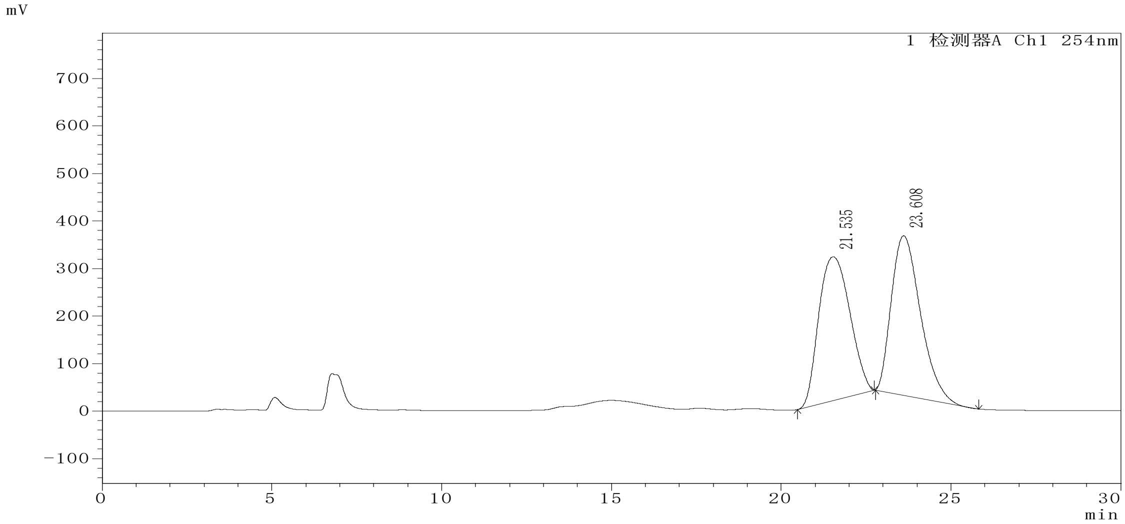

Figure S40. HPLC trace of racemic **3f**

	Retention Time	Area	Area percent
1	21.535	19324995	49.116
2	23.608	20020593	50.884

Total		39345588	100.000

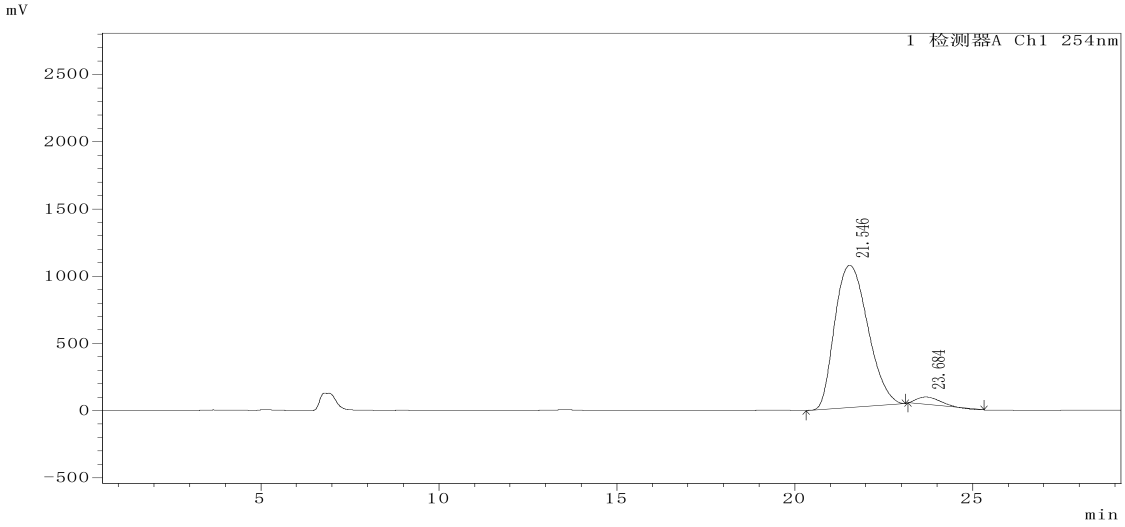

Figure S41. HPLC trace of enantiomeric **3f**

	Retention Time	Area	Area percent
1	21.546	68383306	96.510
2	23.684	2472912	3.490
Total		70856218	100.000

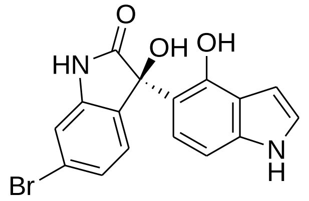

3g

6-bromo-3-hydroxy-3-(4-hydroxy-1*H*-indol-5-yl)indolin-2-one

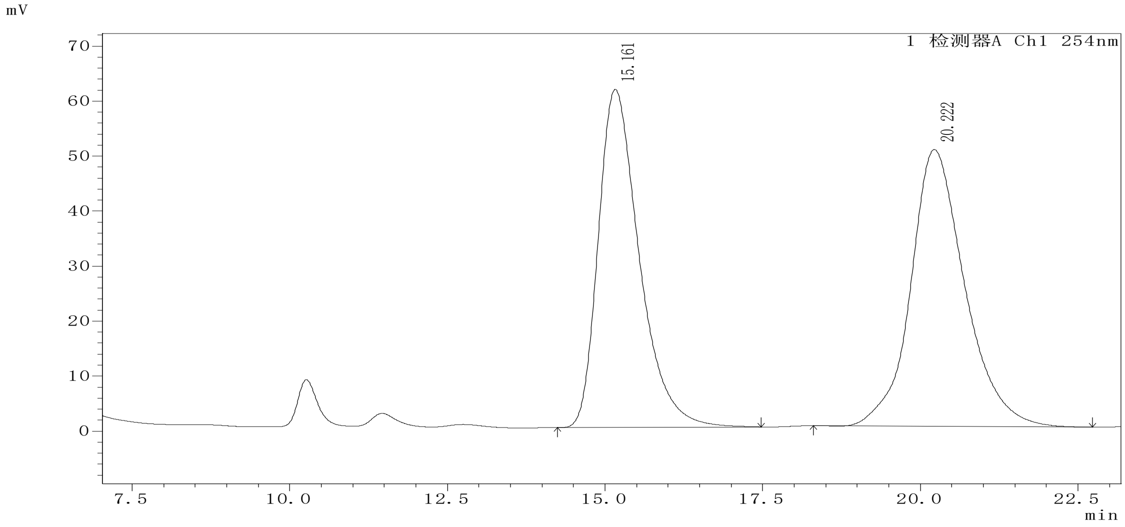

Figure S42. HPLC trace of racemic **3g**

	Retention Time	Area	Area percent
1	15.161	2783746	48.098
2	20.222	3003897	51.902
Total		5787643	100.000

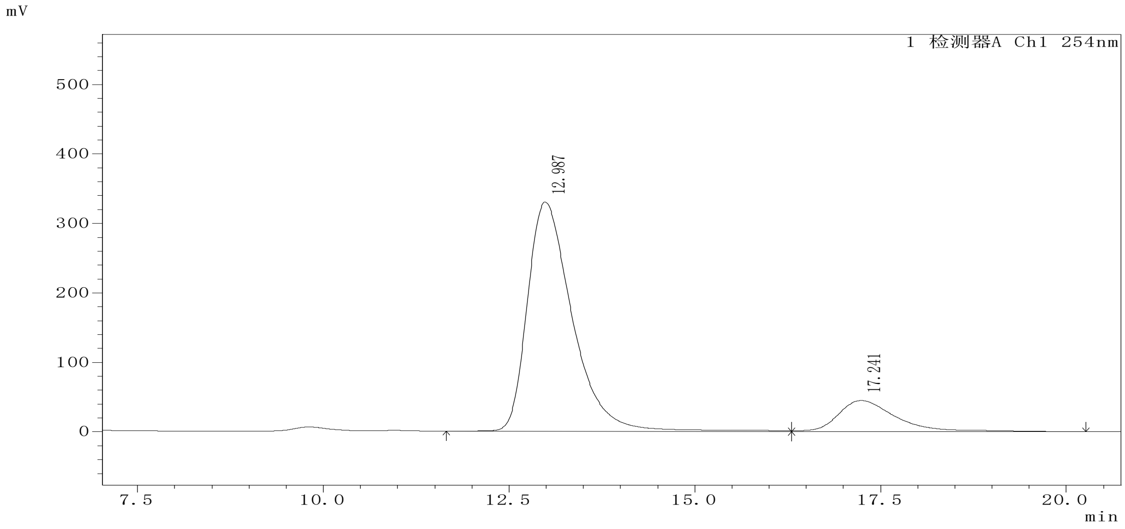

Figure S43. HPLC trace of enantiomeric **3g**

	Retention Time	Area	Area percent
1	12.987	13937111	85.373
2	17.241	2387911	14.627
Total		16325022	100.000

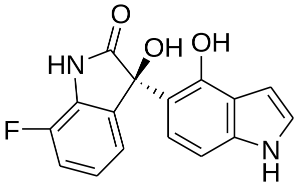

3h

7-fluoro-3-hydroxy-3-(4-hydroxy-1*H*-indol-5-yl)indolin-2-one

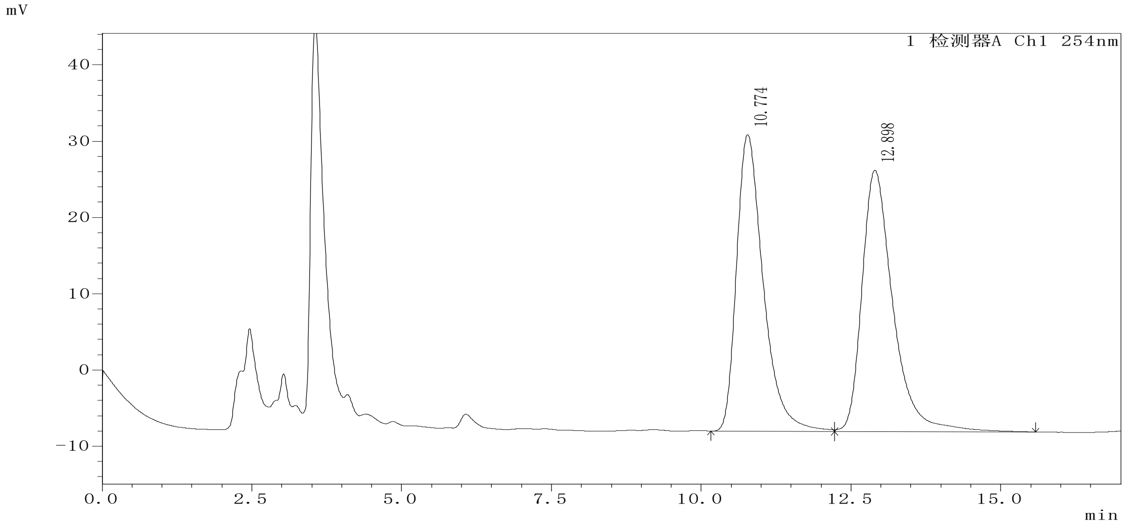

Figure S44. HPLC trace of racemic **3h**

	Retention Time	Area	Area percent
1	10.774	1205113	49.367
2	12.898	1236023	50.633
Total		2441136	100.000

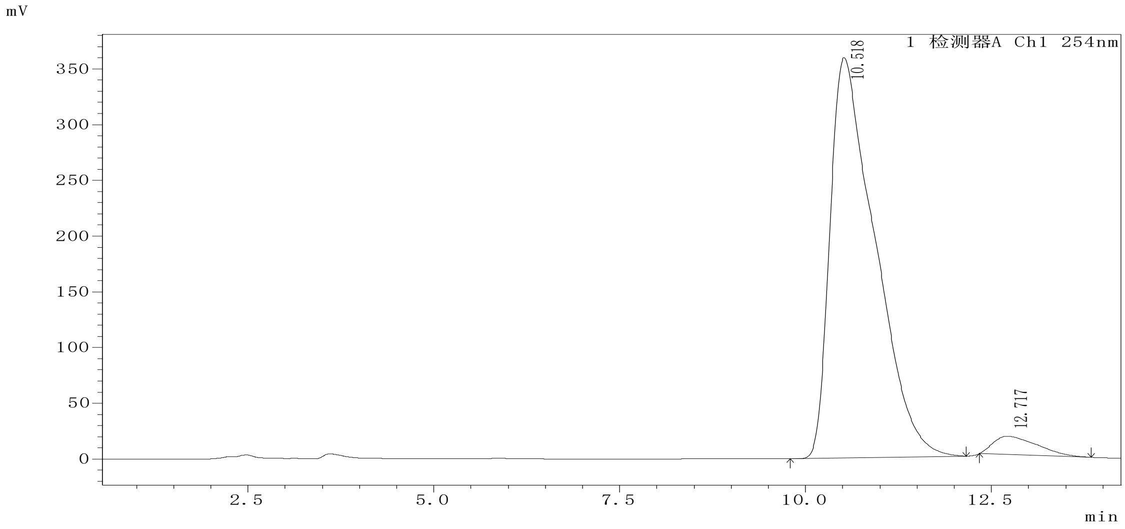

Figure S45. HPLC trace of enantiomeric **3h**

	Retention Time	Area	Area percent
1	10.518	1510826	95.767
2	12.717	667741	4.233
Total		15776001	100.000

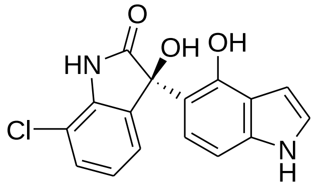

3i

7-chloro-3-hydroxy-3-(4-hydroxy-1*H*-indol-5-yl)indolin-2-one

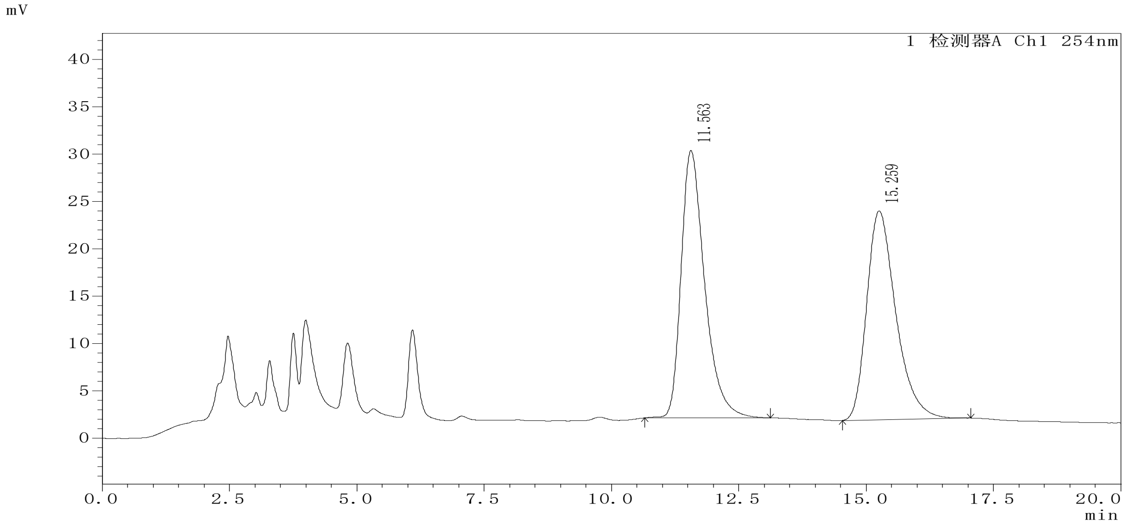

Figure S46. HPLC trace of racemic **3i**

	Retention Time	Area	Area percent
1	11.563	910202	50.854
2	15.259	879644	49.146
Total		1789847	100.000

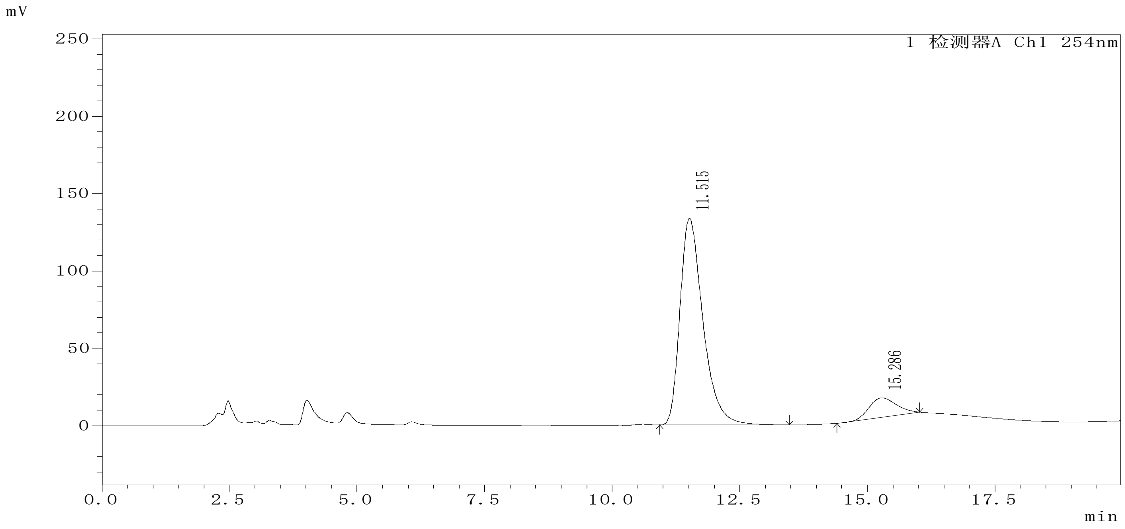

Figure S47. HPLC trace of enantiomeric **3i.**

	Retention Time	Area	Area percent
1	11.515	4189102	91.617
2	15.286	383315	8.383
Total		4572417	100.000

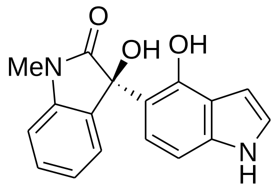

3j

3-hydroxy-3-(4-hydroxy-1*H*-indol-5-yl)-1-methylindolin-2-one

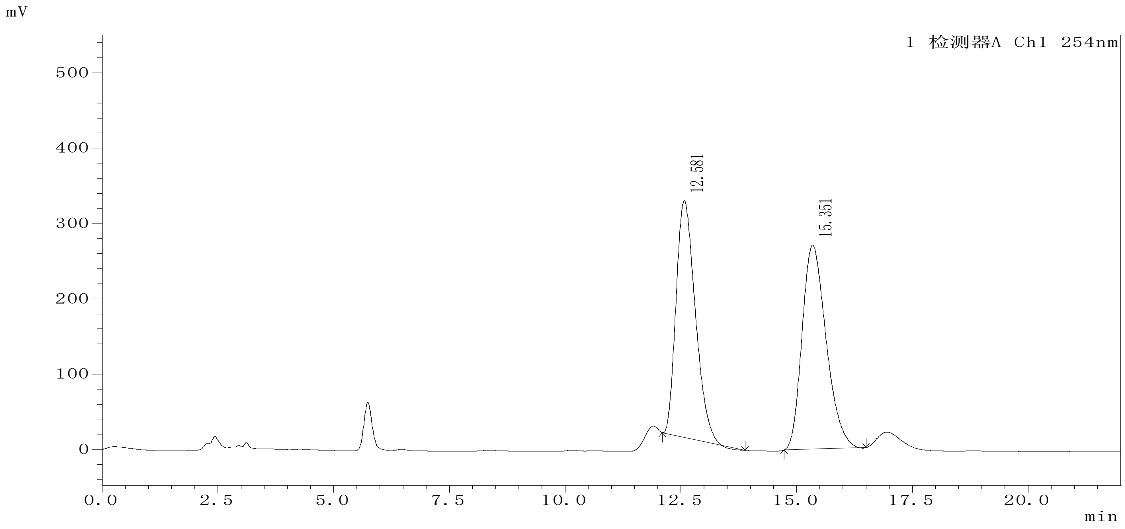

Figure S48. HPLC trace of racemic **3j**

	Retention Time	Area	Area percent
1	12.581	9100714	48.112
2	15.351	9814899	51.888
Total		18915613	100.000

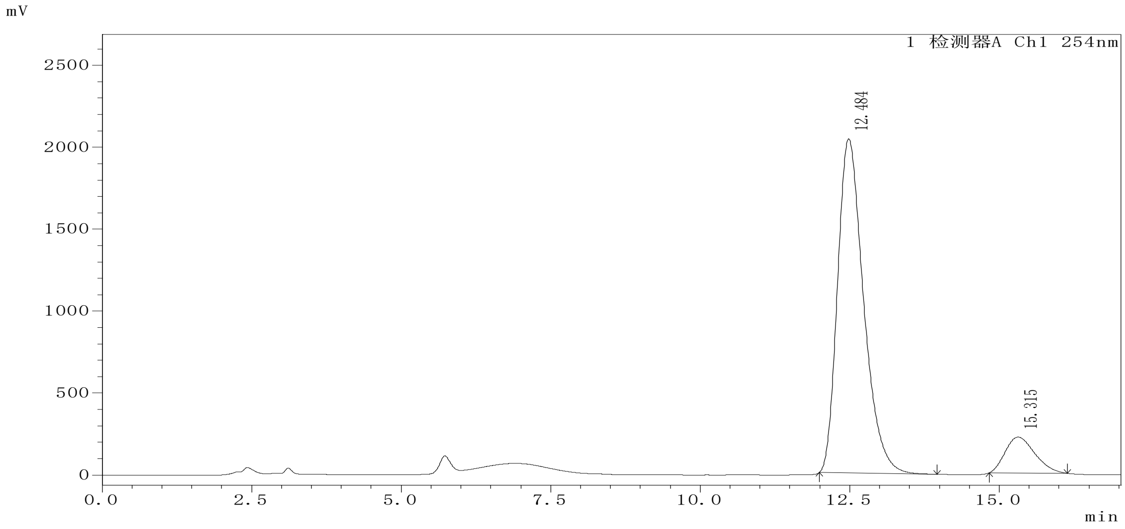

Figure S49. HPLC trace of enantiomeric **3j**

	Retention Time	Area	Area percent
1	12.484	61863000	89.096
2	15.315	7570708	10.904
Total		69433708	100.000

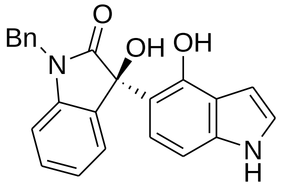

3k

1-benzyl-3-hydroxy-3-(4-hydroxy-1*H*-indol-5-yl)indolin-2-one

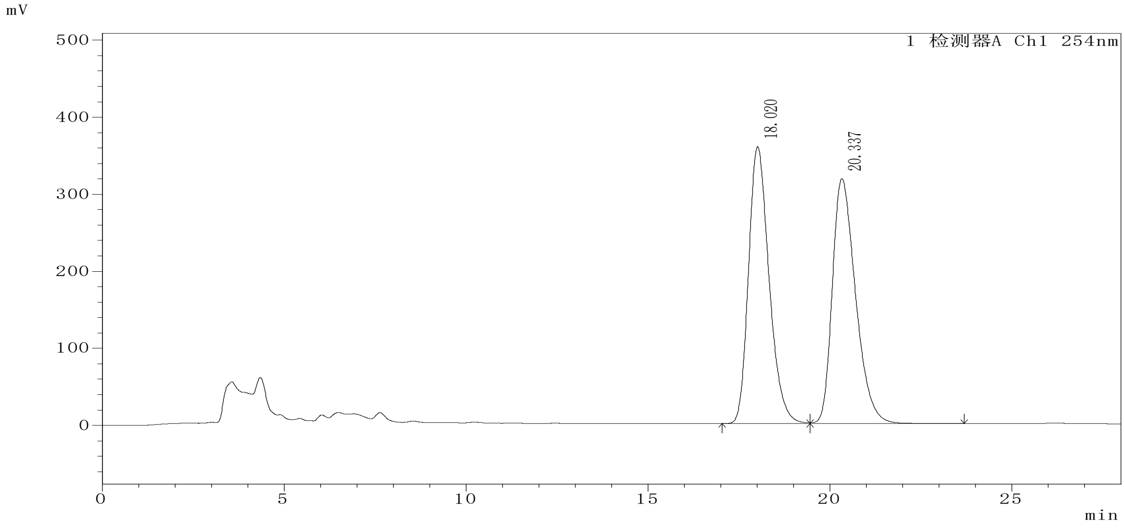

Figure S50. HPLC trace of racemic **3k**

	Retention Time	Area	Area percent
1	18.020	14120921	49.961
2	20.337	14143189	50.039
Total		28264110	100.000

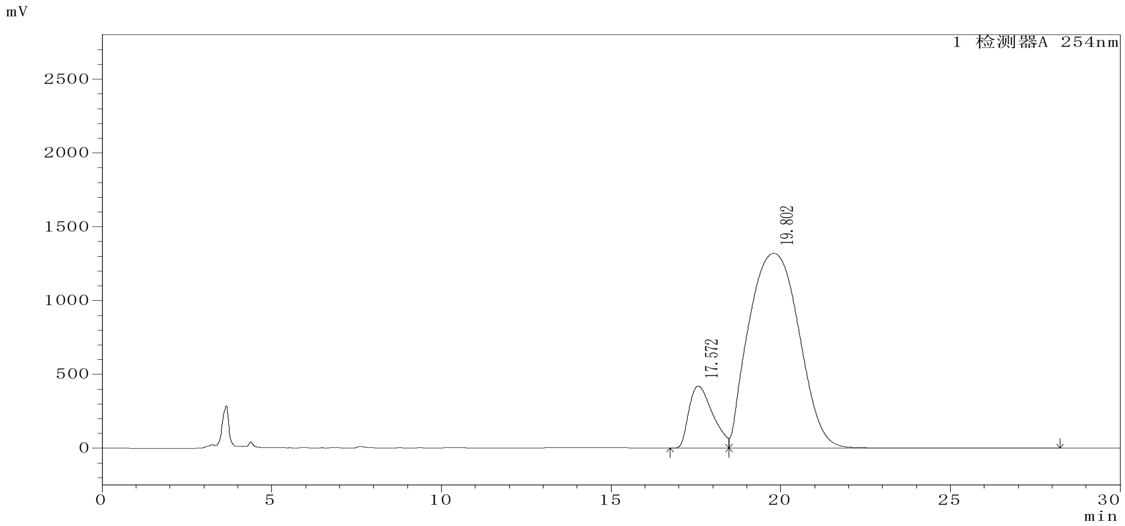

Figure S51. HPLC trace of enantiomeric **3k**

	Retention Time	Area	Area percent
1	17.572	20522224	13.092
2	19.802	136227493	86.908
Total		156749716	100.000

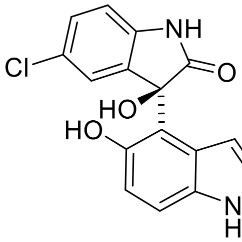

4

5-chloro-3-hydroxy-3-(5-hydroxy-1*H*-indol-4-yl)indolin-2-one

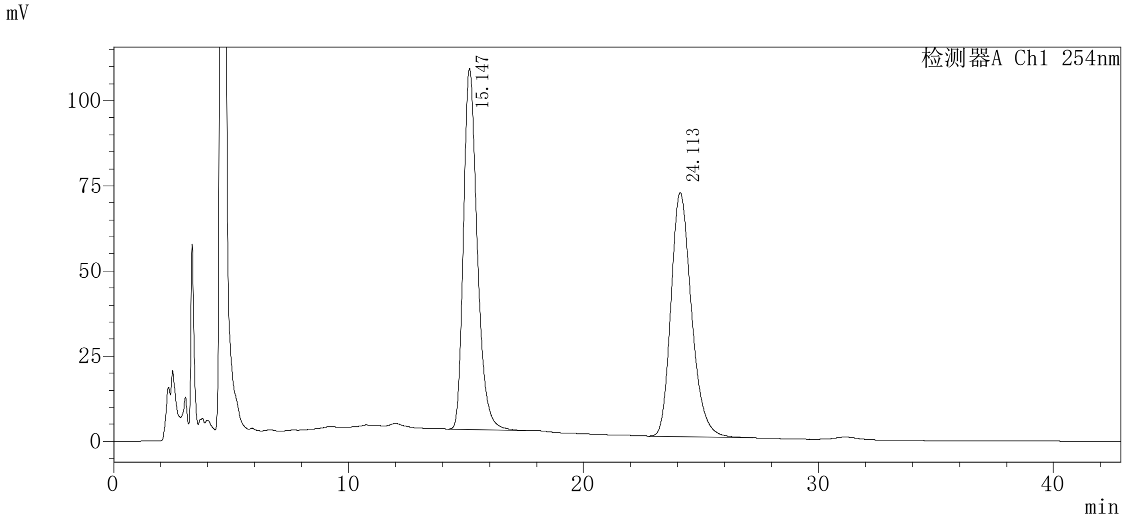

Figure S52. HPLC trace of racemic **4**

	Retention Time	Area	Area percent
1	15.147	4328379	49.901
2	24.113	4345486	50.099
Total		8673865	100.000

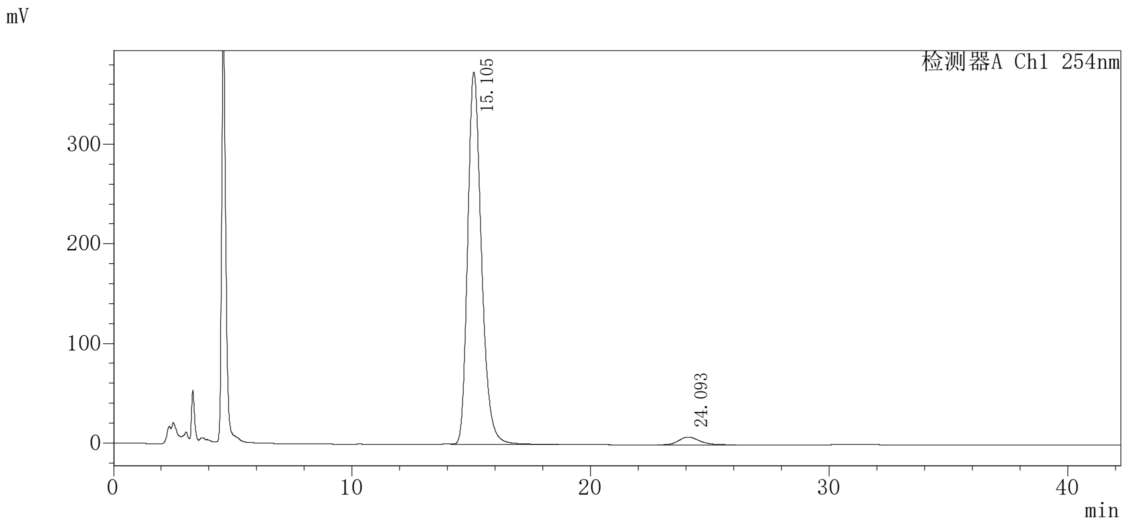

Figure S53. HPLC trace of enantiomeric **4**

	Retention Time	Area	Area percent
1	15.105	14958260	96.929
2	24.093	473877	3.071
Total		15432137	100.000

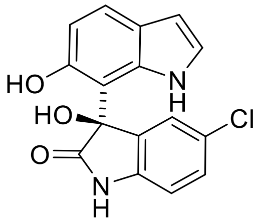

5

5-chloro-3-hydroxy-3-(6-hydroxy-1*H*-indol-7-yl)indolin-2-one

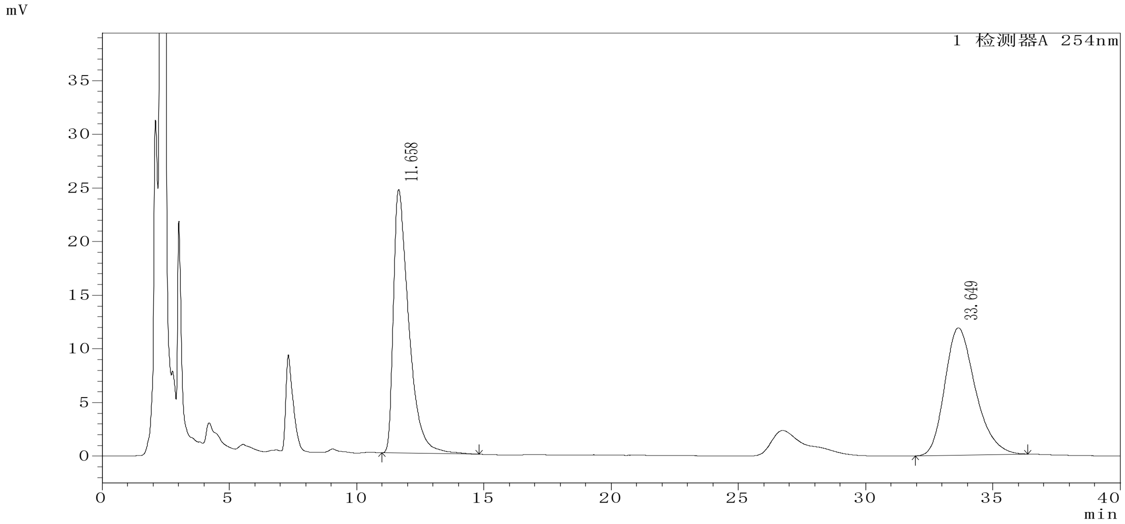

Figure S54. HPLC trace of racemic **5**

	Retention Time	Area	Area percent
1	11.658	1032651	50.966
2	33.649	993490	49.034
Total		2026141	100.000

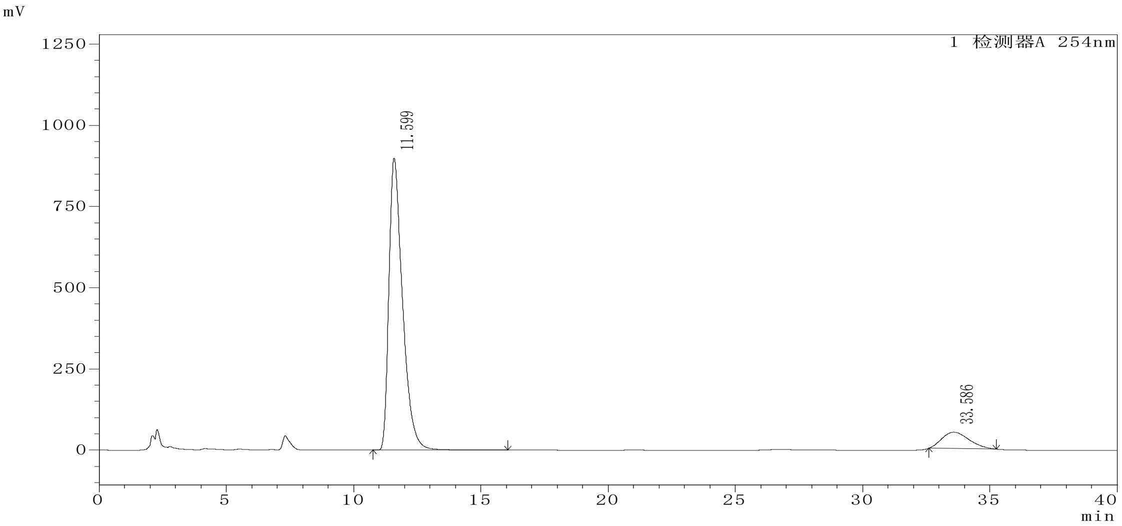

Figure S55. HPLC trace of enantiomeric **5**

	Retention Time	Area	Area percent
1	11.599	33470446	89.918
2	33.586	3752836	10.082
Total		37223281	100.000

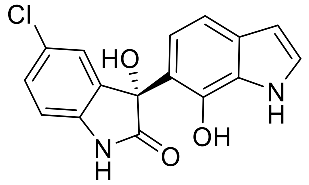

6

5-chloro-3-hydroxy-3-(7-hydroxy-1*H*-indol-6-yl)indolin-2-one

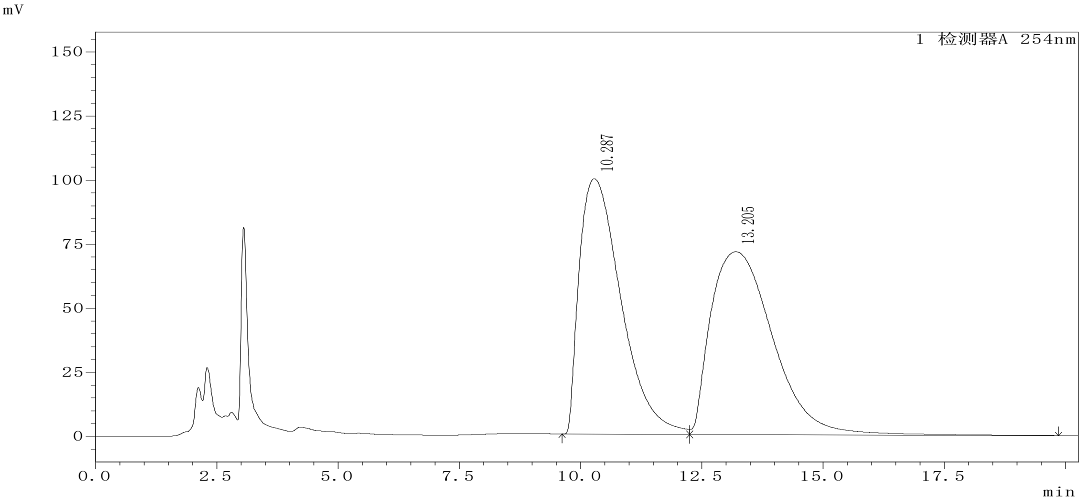

Figure S56. HPLC trace of racemic **6**

	Retention Time	Area	Area percent
1	10.287	5987541	48.453
2	13.205	6369964	51.547
Total		12357506	100.000

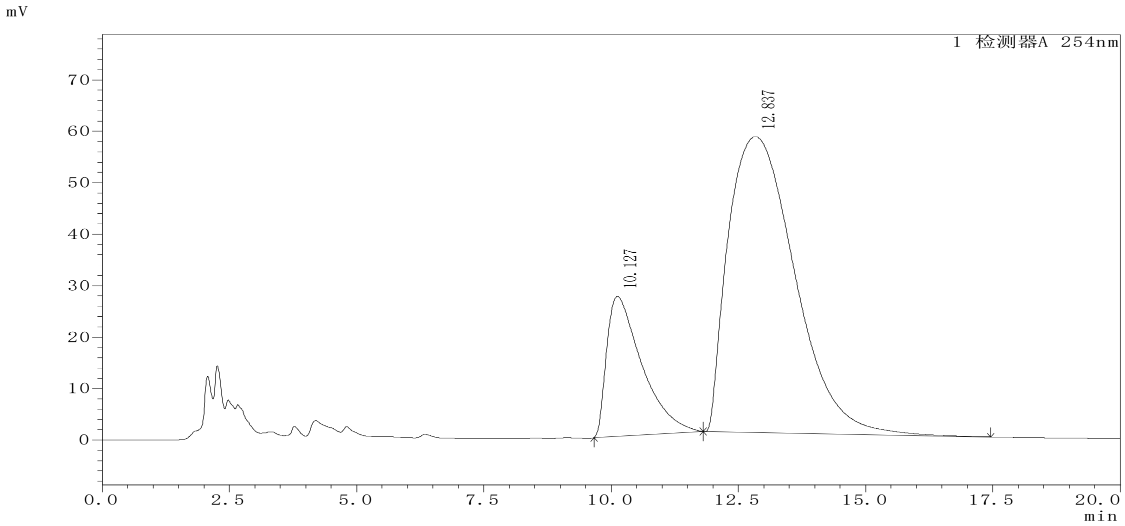

Figure S57. HPLC trace of enantiomeric **6**

	Retention Time	Area	Area percent
1	10.127	1311402	19.986
2	12.837	5250113	80.014
Total		6561515	100.000
